# Correspondence of MRI and nTMS With EDSS in Multiple Sclerosis: Longitudinal Follow‐Up Study

**DOI:** 10.1002/acn3.70041

**Published:** 2025-04-17

**Authors:** Antonia Bralić, Sanda Pavelin, Nikolina Pleić, Joško Šoda, Krešimir Dolić, Anita Markotić, Ana Ćurković Katić, Angela Mastelić, Nikolina Režić Mužinić, Jasna Duranović, Zlatko Kljajić, Ivona Stipica Safić, Zoran Đogaš, Maja Rogić Vidaković

**Affiliations:** ^1^ Department of Interventional and Diagnostic Radiology University Hospital of Split Split Croatia; ^2^ Department of Neurology University Hospital of Split Split Croatia; ^3^ Department of Medical Biology, School of Medicine University of Split Split Croatia; ^4^ Signal Processing, Analysis, Advanced Diagnostics Research and Education Laboratory (SPAADREL), Faculty of Maritime Studies, Department for Marine Electrical Engineering and Information Technologies University of Split Split Croatia; ^5^ Department of Radiology, School of Medicine University of Split Split Croatia; ^6^ University Department of Health Studies University of Split Split Croatia; ^7^ Department of Medical Chemistry and Biochemistry, School of Medicine University of Split Split Croatia; ^8^ Laboratory for Human and Experimental Neurophysiology, Department of Neuroscience, School of Medicine University of Split Split Croatia; ^9^ Faculty of Maritime Studies University of Split Split Croatia; ^10^ Department of Family Medicine, School of Medicine University of Split Split Croatia

**Keywords:** corticospinal tract, motor evoked potentials, multiple sclerosis, relapsing remitting, transcranial magnetic stimulation

## Abstract

**Objectives:**

Considering the characteristics of multiple sclerosis (MS) disease and its impact on motor disability, this study aims to assess the functional integrity of the corticospinal tract by examining motor evoked potentials (MEPs), Expanded Disability Status Scale (EDSS) scores, magnetic resonance imaging (MRI) lesion counts, and psychological and physical status reported by patients with relapsing–remitting MS (RRMS).

**Methods:**

In 23 RRMS subjects (17 in the follow‐up), the corticospinal tract for upper and lower extremity muscles was longitudinally studied over 2 years using navigated transcranial magnetic stimulation (nTMS) and MEP scoring. MRI parameters included lesion detection by applying McDonald's criteria and additional lesion detection of the corticospinal tract. EDSS scoring included evaluation of the EDSS general score, EDSS functional pyramidal score, and EDSS functional pyramidal score for each extremity. Longitudinal analyses of nTMS (MEP), EDSS, and MRI parameters were conducted using linear mixed models with time, sex, age, and disease duration as fixed effects and individual‐specific random intercepts. The correspondence of MRI and nTMS scoring with the EDSS pyramidal scoring was tested using McNemar's or Fisher's exact test.

**Results:**

RRMS patients with altered MEP latency had significantly higher general EDSS scores (*β* = −2.06, *p* = 0.006) and overall pyramid EDSS scores (*β* = −1.96, *p* = 0.002) compared to those with non‐altered MEP latency. This difference was also observed in lower extremity pyramid EDSS scores, with higher scores in the right (*β* = −1.70, *p* = 0.001) and left leg (*β* = −1.50, *p* = 0.032) in the altered MEP latency group. RRMS patients with altered MEP latency had significantly more lesions in the corpus callosum (*β* = 2.38, *p* = 0.03) compared to subjects with non‐altered MEP latency findings. The correspondence of MRI and nTMS (MEP latency) with EDSS scoring was confirmed.

**Interpretation:**

RRMS subjects with altered MEP latency findings (prolonged MEP latency or absent MEP response) compared to subjects with non‐altered MEP latency findings, had higher EDSS scores in lower extremity muscles as well as a higher number of lesions in the corpus callosum. This is the first longitudinal nTMS study to perform four‐limb cortical mapping of corticospinal tract integrity in RRMS. The study opens perspectives for the nTMS as an objective method for longitudinally assessing MS motor disability.

**Trial Registration:**

ClinicalTrials.gov identifier: NCT04604041

## 1 Introduction

Multiple sclerosis (MS) is an inflammatory autoimmune disease of the central nervous system (CNS), of still unknown cause, characterized by demyelinating white matter lesions and neuronal degeneration, which causes various symptoms (motor and sensory dysfunctions, cognitive impairment, mood disorders, fatigue), clinical manifestations and disease progression [1]. The prevalence of MS in the world ranges from 5 to 300 per 100,000 people and affects women more often (distribution between women and men 3:1) [2].

The diagnosis of MS is made based on laboratory findings (e.g., specific bands of immunoglobulins of individual lymphocyte clones in the cerebrospinal fluid) and radiological findings (e.g., magnetic resonance imaging (MRI) ≥ 1.5 T or 3T, T2 lesions in brain and spinal cord, gadolinium‐enhancing lesions) including the application of the 2017 McDonald criteria and the 2021 MAGNIMS‐CMSC‐NAIMS recommendations [3, 4]. The clinical status of disability is expressed through the Expanded Disability Status Scale (EDSS) [5]. Various quantitative measures derived from conventional and advanced MRI methods have been proposed as prognostic biomarkers for MS. However, correlations between different MRI indicators and EDSS are not satisfactory, and no single definitive MRI measure is used as a comprehensive prognostic imaging biomarker for MS [6, 7, 8].

Pathophysiological correlates and their relationship with clinical findings and symptoms are still not fully elucidated, which suggests the need for more precise analyses and the discovery of immunological markers and other paraclinical/subclinical markers that could identify the pathological events involved with MS in different stages of the disease [9, 10]. Most of the clinical symptoms typical of MS are related to the altered generation and transmission of signals in the CNS. Pathological signaling can result from various mechanisms, including demyelination or a localized block in impulse conduction due to axonal damage [11]. Evoked potentials (EP) represent neurophysiological measures of signal conduction in the CNS in vivo. Studies indicate that EPs are predictive of MS disease course and may help to detect MS patients at high risk of progression [11]. EPs can detect deterioration and improvement in impulse propagation, independent of the mechanism causing the change, and are, therefore, good candidates for biomarkers of neurophysiological status. Because of its high sensitivity for subclinical lesions and relatively high specificity, MRI has largely replaced EP in standard clinical examinations due to MRI dissemination in time and space in patients with typical symptoms of a demyelinating event [12]. However, the ability of EP to detect even subclinical lesions of pathways, not well investigated in routine MRI evaluations (optic nerve and spinal cord‐corticospinal tract [CST]), has been demonstrated previously [11]. In patients with PPMS, spinal syndromes often prevail, and visual evoked potentials (VEPs) are frequently abnormal (in about 90%) even without corresponding clinical signs and therefore have diagnostic value [13]. The prognostic power of EP is more pronounced in the early stages of RRMS and PPMS. The relationship increases with the length of the observation period, and it has been shown that multimodal EPs (i.e., somatosensory EPs—SEPs, VEPs, and MEPs) recorded as baseline at diagnosis, correlate with EDSS even after 20 years [14]. The relationship between structural measures of conventional MRI (brain atrophy, development of hypointense T1‐lesions) and disease progression is moderate, which indicates that EP measures might be better related to clinical disability compared to structural data obtained by classical MRI.

Examining corticospinal excitability as a marker of functional integrity of the primary motor cortex (M1) and the corticospinal pathway using electric‐field navigated transcranial magnetic stimulation (nTMS) could help further understanding of the underlying pathophysiological mechanisms of the motor pathways in MS [15, 16, 17]. Concerning the TMS clinical use and interpretation of MEPs in the monitoring of the CST integrity in MS, TMS guidelines were previously recommended by Fernández et al. in the year 2013 [18], mainly referring to the TMS application without navigation, including the magnetic stimulator connected to a standard EMG unit, and less to line navigated TMS implementations (TMS with line navigation and e‐field navigation techniques). Line‐navigated TMS is performed without visualization of the spot of maximal stimulation if there is a slight coil tilt and is susceptible to errors when the coil is not held continuously tangentially against the head [19, 20]. Electric‐field nTMS computes online the maximum e‐field where the cortex is optimally stimulated, and it considers the geometry of the head, the magnetic coil shape, location, orientation, individual head shape, size, and the orientation of the cortical folds. Therefore, the use of e‐field nTMS assessment might improve the accuracy of CST integrity testing by providing a more objective correspondence of the TMS (MEP) and EDSS classifications [17].

With the application of a single pulse, the TMS method enables the investigation of several neurophysiological measures of the excitability of the motor corticospinal pathway, such as motor threshold (MT), MEP latency, MEP amplitude, and the duration of the cortical silent period (CSP) [21, 22]. Recent findings suggest the use of TMS as a subclinical test that could help identify biomarkers of MS disease [23, 24] and represent a biomarker for monitoring MS disability [23, 25, 26].

Current data suggest an association between the pathophysiological mechanisms of MS (demyelination and loss of axons) and TMS neurophysiological measures (e.g., lower amplitudes and longer latencies of MEP responses from upper and lower limb muscles, elevated MT at rest), and changes in specific neurophysiological measures of excitation and inhibition [9, 22, 25]. Furthermore, changes in cortical excitatory and inhibitory processes in MS assessed with TMS appear to be evident in early disease progression, during relapse, and later during disease progression [10, 13, 27, 28, 29, 30]. Also, changes in neurophysiological TMS measures are associated with clinical characteristics of MS [5, 9, 10, 13, 16, 25, 26, 27, 28, 29, 30, 31]. The clinical status of disability, expressed through the EDSS score [5] or dexterity and ambulation scores is associated with an increased MT, lower MEP amplitude, prolonged MEP latency, decreased slope of the MEP amplitude IO (input–output) curve, and prolonged CSP [16, 22, 25, 26]. To date, longitudinal studies demonstrated that MEP latency displays the most specific correlation and highest predictive value for disability progression [18] and sensitivity change over time [28], with MEPs of the lower limb having the highest significance in discriminating progressive from relapsing–remitting MS disease [13, 26, 28, 31]. Further, multimodal EPs, including tibial MEP latency and SEP latencies, proved to have the greatest prognostic values; [30] however, there is limited data on the influence of approved MS treatments on EPs. Several studies reported improvements in VEP and SEP scores 1 year after immunomodulatory drug treatments with no significant effect on the MEP scores [32, 33, 34]. One recent study reported significant improvement in tibial SEP latencies with a similar trend for MEP latencies 1 year after autologous hematopoietic stem cell transplantation in relapsing–remitting MS, advocating the use of EPs as a tool for assessing clinical response to MS treatment [35].

This nTMS study aimed to investigate MEP scores after 2 years of follow‐up as an objective measure of signal transmission in the CST in RRMS subjects in addition to EDSS, MRI lesion count, and patient‐derived scores on physical and psychological status.

## 2 Materials and Methods

### 2.1 Participants

The study enrolled 23 relapsing–remitting MS (adult RRMS) patients in a 2‐year follow‐up, with a mean age of 42 ± 9 [5, 9, 10, 13, 24, 25, 26, 27, 28, 29, 30, 31, 32, 33, 34, 35, 36, 37, 38, 39, 40, 41, 42, 43, 44, 45, 46, 47, 48] at baseline. The first assessment was performed in 2022, while the second nTMS assessment was conducted in 2024. Participants were recruited from the Department of Neurology University Hospital of Split, Croatia. Radiological examinations were performed 2–7 days before nTMS and neurological examination, while neurological and neurophysiological assessments with nTMS were performed on the same day. Inclusion criteria were as follows: age ≥ 18 years, diagnosis of relapsing–remitting MS at least 12 months before inclusion in the study (first enrollment, year 2022); the absence of clinical or neuroradiological disease activity at least in the 3 months before assessment; patients treated with the same neuromodulatory drug (teriflunomide) medication for ≥ 12 months (first enrollment, year 2022); and without rehabilitation in the previous 3 months before study beginning. Exclusion criteria were the presence of comorbidity affecting ambulation; history of diseases of the central or peripheral nervous system (other than relapsing–remitting MS); history of psychiatric diseases, drug or alcohol abuse; contraindication to nTMS (history of epilepsy, recent brain surgery or trauma, previous stroke, pregnancy, the presence of metallic implant, denture or cardiac pacemaker).

### 2.2 Clinical Evaluation—Radiology and Neurology Assessment

Neurological examination and medical history included the following measures: the EDSS (general) score, EDSS functional pyramidal score, and EDSS functional pyramidal score for each extremity, MS disease duration, drug intake duration, and comorbidities other than MS. The electroneurographic (ENG) assessment was performed before nTMS to exclude peripheral neurological events (Medelec‐Synergy instrument, Oxford Instrument Co., Surrey, UK).

Radiological magnetic resonance imaging (MRI) scans were acquired on a 1.5 T MR system (Avanto, Siemens, Germany) using a 12‐channel phased array head coil. The sequences included in the brain scan protocol were 3D T1‐weighted images, axial T2‐weighted images, and fluid‐attenuated inversion recovery (FLAIR) images in the axial and sagittal plane, all used to identify brain lesions. Spinal cord sequences included sagittal T2‐weighted images, sagittal turbo inversion recovery magnitude (TIRM) images, and axial T2 med images from C1 to C7 vertebral levels. MRIs were analyzed using a Syngo.via software (Siemens Healthcare, Forchheim, Germany). MRIs were analyzed by two senior radiology neuroradiologists (AB and KD). Using the T2, FLAIR, TIRM, and T2 med images, specific locations of the CST were visually examined, including subcortical white matter in the primary motor cortex (CST‐M1), capsula interna, cerebral peduncles and ventral parts of the midbrain and pons (CST‐M2) and ventral and lateral parts of the cervical spinal cord (CST‐M3). For each subject, it was checked whether they had a lesion in any listed locations (CST‐M1, CST‐M2, CST‐M3), the number of lesions, and whether the lesion was located on the left or right side. The McDonald's criteria were consulted for the lesion count (cortical, juxtacortical, periventricular, infratentorial, spinal cord, corpus callosum) for the individual subject [3, 4, 31].

MRI images (DICOM format) were used for the 3D reconstruction of individual brain anatomy (3D optical tracking unit of the manufacturer Polaris Vicra) with EF‐nTMS technique (Nexstim NBS System 4 of the manufacturer Nexstim Plc., Helsinki, Finland).

### 2.3 Patient‐Derived Measures of Psychological and Physical Status

The patient‐derived measures of psychological and physical disability were as follows: the MS Impact Scale (MS Impact Scale) (MSIS‐29) [36, 37], and the Depression, Anxiety, and Stress Scale‐21 (DASS‐21) [37, 38].

### 2.4 Motor Evoked Potentials (MEP)

**FIGURE 1:**
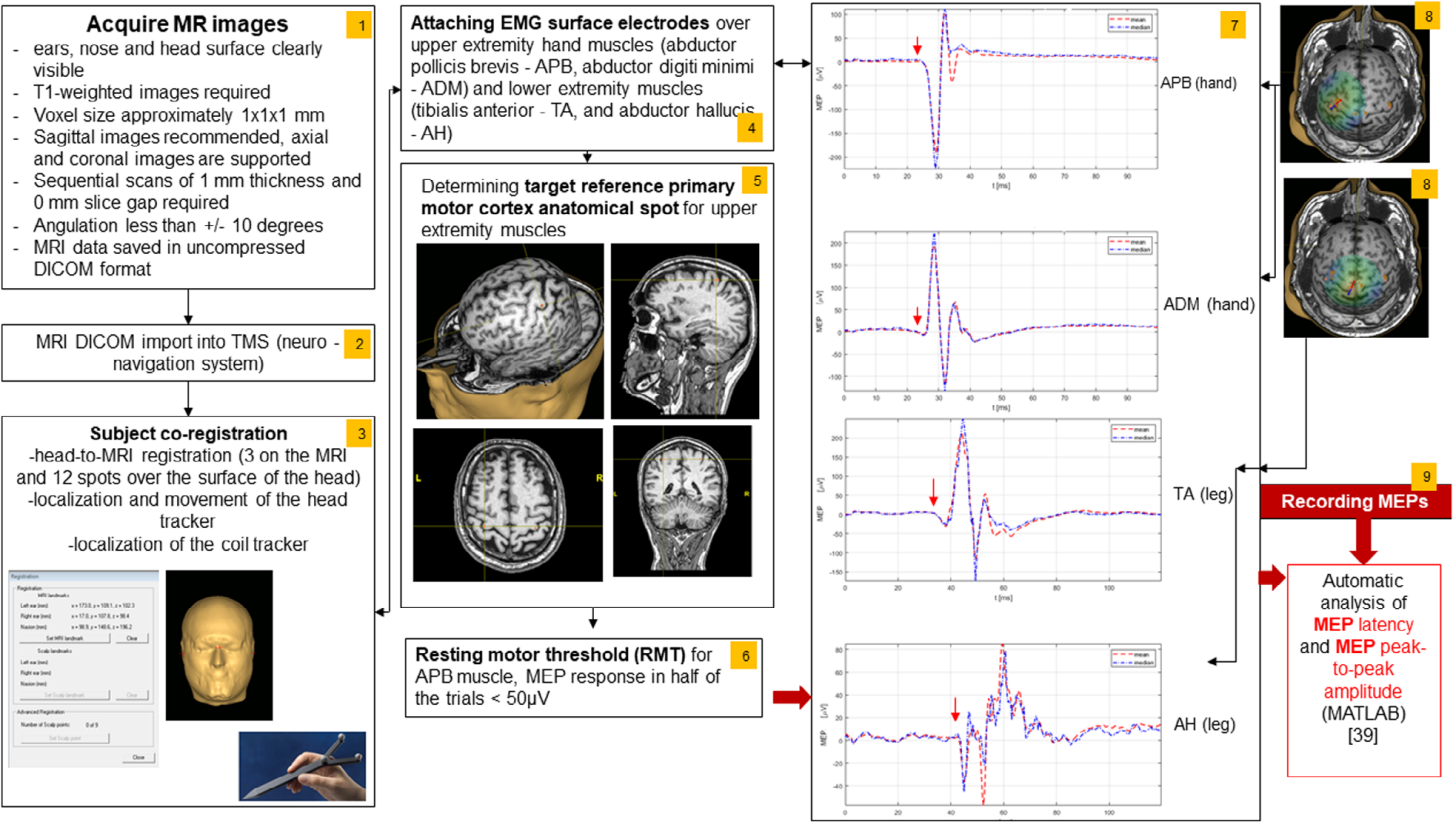
Schematic procedure for assessment of corticospinal tract integrity with nTMS. 1. Acquisition of MRI images for the nTMS reconstruction; 2. Import of DICOM images into nTMS; 3. Subject co‐registration; 4. Attachment of electrodes over target muscles for motor evoked potential (MEP) recording; 5. Detection of anatomical referent spot over the primary motor cortex corresponding to the representation of the upper extremity muscle for resting motor threshold (RMT) evaluation; 6. RMT evaluation; 7. Examples of MEPs (mean and median) were recorded from target muscles of the upper (APB, ADM) and lower (TA, AH) extremity muscles. Red arrows indicate the MEP latency onset.; 8. TMS coil positioned over the left primary motor cortex for upper and lower extremity muscles; 9. Automatic analysis of MEP signals performed by Šoda et al. [39]

Figure 1 presents the schematic procedure for mapping the M1 and finding the hot spot of the upper and lower extremity muscles with an electric‐field nTMS system (Nexstim NBS System 4 of the manufacturer Nexstim Plc., Helsinki, Finland). Before the nTMS experiment, each subject's head MRI was used for the 3D reconstruction of individual anatomy (3D optical tracking unit, Polaris Vicra). The MRI is co‐registered with the subject head using the Nexstim forehead tracker tracking system. When the coil rests against the subject head, the electric field is overlaid on the 3D model of the brain. As the coil is moved, the magnitude (V/m) and orientation of the electric‐field relative to the cortex are dynamically calculated and displayed in real time. The maximum strength of the electric field measured 25 mm below the coil in a spherical conductor model representing the human head was 172 V/m. The magnetic stimulation was delivered using a biphasic magnetic coil generating a single biphasic pulse of a length of 289 μs. The eight‐shaped coil with an inner winding diameter of 50 mm and an outer winding diameter of 70 mm was placed tangentially to the subject's skull over the M1. The MEPs were recorded from the upper extremity muscles (abductor pollicis brevis—APB, abductor digiti minimi—ADM) and lower extremity muscles (tibialis anterior—TA, abductor hallucis—AH) with a pair of self‐adhesive surface electrodes (Ambu Neuroline 710, REF 71005‐K/12 of manufacturer Ambu A/S, Denmark) in a belly‐tendon montage. Electrodes were attached to the electrode cable of the Nexstim electromyography (EMG) with a 1.5 mm touch‐proof female safety connector (DIN 42‐802) and connected to a 6‐channel EMG and one common ground EMG amplifier (external module) with TMS‐artifact rejection circuitry. EMG characteristics were 3 kHz, resolution: 0.3 μV, and scale: between −7.5 mV and 7.5 mV. The coil was positioned perpendicular to the central sulcus to ensure a posterior–anterior current direction over the M1. The lowest stimulation intensity used to elicit at least five positive MEP responses out of 10 trials, having peak‐to‐peak amplitudes larger than 50 μV, was defined as the resting motor threshold (RMT) intensity (RMT100%). This strategy was applied in mapping the M1 to find the hot spot for upper and lower extremity muscles (APB, ADM, TA, and AH). When mapping the M1 for the hot spot for lower extremity muscles (TA, AH) and applying 100% intensity of maximal stimulator output, MEP responses could not be elicited in some subjects. In some subjects, on maximal stimulator output of 100%, lower amplitude peak‐to‐peak MEP response with an amplitude of lower than 50 μV could be elicited; in these cases, 5–10 MEP trials were collected. RMT120% intensity was also applied in the follow‐up to record MEPs from the upper extremity muscles but was not used in the statistical analysis. MEP latency and amplitude estimation were performed by MATLAB script (R2021a) using an automatic algorithm [39]. Šoda et al. [39] developed the Squared Hard Threshold Estimator (SHTE), a novel and improved algorithm for MEP latency and amplitude estimation, reliable especially for the MEP signals with peak‐to‐peak amplitudes lower than 100 μV.

### 2.5 Statistical Analysis

Baseline demographic and clinical characteristics of the study participants, as well as their characteristics at the 2‐year follow‐up, are presented in Table 1. Categorical variables, including sex, handedness, education level, working status, use of assistive technology, BMI categories, and smoking status, were reported as absolute and relative frequencies. The *χ* ^2^ goodness‐of‐fit test was used to assess the distribution of categorical variables at both time points.

**TABLE 1:** Demographic characteristics of the participants in the baseline and 2‐year follow‐up.

Variable	Baseline	Two‐year follow‐up
	Mean (SD) or median (Q1–Q3) or absolute frequency (relative frequency)	Mean (SD) or median (Q1–Q3) or absolute frequency (relative frequency)
*Sex*		
Female	14 (60.9%)	8 (47.06%)
Male	9 (39.1%)	9 (52.94%)
*p*	0.297^a^	0.808^a^
*Edinburgh handedness inventory*		
1 (Right‐handed)	21 (91.3%)	15 (88.24%)
2 (Left‐handed)	2 (8.7%)	2 (11.76%)
*p*	**7.44 × 10** ^**−5**^ ^**a**^	**0.002** ^**a**^
*Education*		
1 (elementary school)	2 (8.7%)	2 (11.76%)
2 (high school)	17 (73.9%)	13 (76.47%)
5 (undergraduate university study)	1 (4.3%)	1 (5.88%)
6 (graduate university study)	3 (13%)	1 (5.88%)
*p*	**1.60 × 10** ^**−6**^ ^**a**^	**2.30 × 10** ^**−5**^ ^**a**^
Weight (kg)	77.00 (64–92)	82.8 (19.3)
BMI	25.14 (3.78)	26 (3.39)
*BMI classified*		
Underweight	1 (4.3%)	0 (0%)
Lean	2 (8.7%)	1 (5.88%)
Normal	7 (30.4%)	4 (23.53%)
Overweight	11 (47.8%)	10 (58.82%)
Obese (class I)	2 (8.7%)	2 (11.76%)
*p*	**0.003** ^**a**^	**0.0009** ^**a**^
*Working status*		
Student	1 (4.3%)	0 (0%)
Employed	10 (43.5%)	8 (47.06%)
Unemployed	8 (34.8%)	6 (35.29%)
Temporarily on sick leave (otherwise employed)	1 (4.3%)	0 (0%)
Pensioner (disability pension)	3 (13%)	3 (17.65%)
*p*	**0.005** ^**a**^	**0.005** ^**a**^
*Assistive technology*		
Glasses	14 (60.9%)	10 (58.82%)
None	9 (39.1%)	7 (41.18%)
*p*	0.297^a^	0.467^a^
*Smoker*		
Yes	7 (30.4%)	5 (29.41%)
No	15 (65.2%)	11 (64.71%)
Occasionally	1 (4.3%)	1 (5.88%)
*p*	**0.002** ^**a**^	**0.011** ^**a**^

*Note:* A BMI below 18.5 is considered underweight, A BMI less than 20 may be reported as lean, a BMI of 25 or more is considered overweight, a BMI of 30 or more is considered obese (class I). Statistically significant *p*‐values are indicated in bold.

Abbreviation: BMI, body mass index.

^a^ *χ* ^2^, Goodness of fit test.

Table 2 presents TMS (MEP scores), EDSS, and psychological‐physical status scores on DASS‐21, and MSIS‐29. The EDSS general score and pyramid scores were reported as medians with interquartile ranges (Q1–Q3), while DASS‐21 and MSIS‐29 scores were presented as means with standard deviations (SD). Additionally, nTMS measures, including RMT (%), MEP latency (ms), and MEP amplitude (μV), were reported as means with SD for the APB, ADM, TA, and AH muscles for nTMS stimulation of both hemispheres.

**TABLE 2:** nTMS scores (intensity, MEP latency, MEP amplitude), EDSS pyramid score, and psychological‐physical status scores (DASS‐21, MSIS‐29) at baseline and 2‐year follow‐up in all subjects.

	Baseline	Two‐year follow‐up
	Median (Q1–Q3)	Median (Q1–Q3)
EDSS (general score)	2.5 (0.5–3.5)	2 (1–3.5)
EDSS pyramid score	2 (0.5–3)	2 (1–3)
EDSS pyramid score right leg	1 (0–2.25)	1 (1–2)
EDSS pyramid score left leg	1 (0–2.25)	1.5 (0–2)
EDSS pyramid score right arm	0 (0–0)	0 (0–1)
EDSS pyramid score left arm	0 (0–0)	0 (0–1.2)

	Baseline	Two‐year follow‐up
	Mean (SD)	Mean (SD)
DASS‐21 (depression score)	3.39 (3.58)	4.82 (6.19)
DASS‐21 (anxiety score)	4.35 (4.23)	4.41 (4.72)
DASS‐21 (stress score)	6.30 (5.45)	5.71 (6.03)
MSIS‐29 (physical score)	37 (16.1)	42.6 (20.3)
MSIS‐29 (psychological score)	18.7 (8.44)	21.7 (10.8)

nTMS left hemisphere stimulation (measures)	Baseline	Two‐year follow‐up
	Mean (SD)	Mean (SD)
APB RMT %	40.3 (9.42)	40.1 (8.83)
APB MEP latency (ms)	24.1 (3.70)	24.6 (1.90)
APB MEP amplitude (μV)	276.07 (172.45)	249.92 (131.19)
ADM RMT %	42.3 (12.1)	41.3 (10.1)
ADM MEP latency (ms)	23.9 (3.12)	23.9 (1.72)
ADM MEP amplitude (μV)	162.70 (86.77)	157.40 (78.15)
TA RMT %	81.1 (15.6)	81 (16.7)
TA MEP latency (ms)	36.1 (7.09)	32.6 (2.61)
TA MEP amplitude (μV)	101.57 (54.23)	114.56 (92.69)
AH RMT %	78.2 (16.4)	74.3 (15.1)
AH MEP latency (ms)	47 (5.01)	45.3 (4.33)
AH MEP amplitude (μV)	253.52 (141.33)	237.04 (139.02)

nTMS right hemisphere stimulation (measures)	Baseline	Two‐year follow‐up
	Mean (SD)	Mean (SD)
APB RMT %	47 (14.2)	41.4 (6.47)
APB MEP latency (ms)	23.8 (2.72)	27.1 (9.16)
APB MEP amplitude (μV)	228.83 (224.13)	216.78 (103.25)
ADM RMT %	48.9 (14.5)	45.2 (16.3)
ADM MEP latency (ms)	24.1 (2.85)	26.3 (8.23)
ADM MEP amplitude (μV)	204.10 (142.06)	209.01 (215.42)
TA RMT %	82.6 (16.1)	87.8 (30.9)
TA MEP latency (ms)	34.3 (4.05)	36.1 (5.62)
TA MEP amplitude (μV)	143.03 (150.75)	113.88 (97.27)
AH RMT %	78.7 (16.6)	75 (16.2)
AH MEP latency (ms)	44.9 (4.56)	45.3 (3.87)
AH MEP amplitude (μV)	378.96 (267.82)	239.89 (152.01)

Table 3 further details nTMS measures in a subgroup of RRMS patients with altered MEP latency at baseline. The same neurophysiological parameters (RMT %, MEP latency, and MEP amplitude) were analyzed for nTMS stimulation of both hemispheres, allowing for the assessment of potential changes over the 2 years. All nTMS measures were reported as means with SD. A longitudinal analysis of the TMS, EDSS, and MRI parameters in observed patients was performed using a linear mixed model (LMM) in which the mentioned parameters were a dependent variable and time was and modeled as a fixed effect (baseline vs. follow‐up). Sex (coded as 1 = female, 2 = male), age, and MS disease duration were included as additional covariates. A random intercept was included in each LMM using individual identification (ID) to account for individual‐specific variability, allowing each patient to have a unique baseline measurement. This approach captured intra‐individual correlations in repeated measures, improving model accuracy and preventing bias in the estimation of fixed effects. Unlike ANOVA, which requires complete data across all time points and would otherwise exclude participants with partial data, the LMM approach utilizes all available data points, preserving the sample size, modeling individual differences and complex temporal effects more flexibly, and providing greater reliability and validity in longitudinal analyses by including data from all 23 subjects. All LMM analyses were first performed on the total sample (*n* = 23) with fixed effects of time, sex, age, and MS disease duration. The RRMS patients were subsequently grouped, based on MEP latency findings [16, 21, 22], into non‐altered and altered MEP (prolonged MEP latency or absent MEP response) latency findings groups. Following this, all LMM analyses were also conducted on the total sample with an additional fixed effect for this grouping. For the MRI parameters, which represented count data (i.e., the number of lesions), the LMM was replaced by a generalized linear mixed model (GLMM) with a Poisson distribution, appropriate for modeling count data. The difference in the correspondence of TMS with EDSS and MRI with EDSS scales between baseline and follow‐up and between MEP findings groups was tested using McNemar's test or Fisher's exact test.

**TABLE 3:** nTMS scores (intensity, MEP latency, MEP amplitude) in RRMS with altered TMS (MEP latency) findings at baseline and 2‐year follow‐up.

nTMS left hemisphere stimulation (measures)	Baseline	Two‐year follow‐up
	Mean (SD)	Mean (SD)
APB RMT %	41.46 (10.29)	41.00 (10.07)
APB MEP latency (ms)	25.14 (4.12)	25.21 (1.68)
APB MEP amplitude (μV)	318.95 (192.71)	280.66 (143.19)
ADM RMT %	44.53 (13.78)	42.68 (11.82)
ADM MEP latency (ms)	24.79 (3.51)	24.50 (1.68)
ADM MEP amplitude (μV)	167.18 (102.03)	142.77 (75.30)
TA RMT %	85.78 (15.05)	86.90 (13.78)
TA MEP latency (ms)	39.39 (7.43)	34.5 (2.14)
TA MEP amplitude (μV)	96.30 (49.46)	140.87 (122.79)
AH RMT %	84.06 (15.06)	76.55 (15.24)
AH MEP latency (ms)	49.08 (5.04)	47.82 (4.09)
AH MEP amplitude (μV)	208.96 (141.18)	229.96 (163.47)

nTMS right hemisphere stimulation (measures)	Baseline	Two‐year follow‐up
	Mean (SD)	Mean (SD)
APB RMT %	49.86 (16.44)	40.9 (6.45)
APB MEP latency (ms)	24.51 (3.12)	29.59 (10.66)
APB MEP amplitude (μV)	178.46 (83.47)	208.89 (95.17)
ADM RMT %	52.26 (16.63)	46.37 (19.93)
ADM MEP latency (ms)	25.10 (2.93)	28.38 (9.68)
ADM MEP amplitude (μV)	210.34 (134.12)	135.05 (69.57)
TA RMT %	89.53 (11.75)	99.36 (31.06)
TA MEP latency (ms)	35.57 (4.67)	38.24 (6.08)
TA MEP amplitude (μV)	147.95 (184.98)	113.91 (121.89)
AH RMT %	87.21 (12.91)	81.54 (15.04)
AH MEP latency (ms)	46.61 (5.22)	46.95 (3.98)
AH MEP amplitude (μV)	260.13 (114.86)	222.097 (130.06)

Abbreviations: μV, microvolts; ADM, abductor digiti minimi; AH, abductor hallucis; APB, abductor pollicis brevis; DASS‐21, Depression, Anxiety, and Stress Scale‐21; EDSS, Expanded Disability Status Scale; ms, milliseconds; MSIS‐29, Multiple Sclerosis Impact Scale‐29; Q1, interquartile 1; Q3, interquartile 3; SD, standard deviation; TA, tibialis anterior.

**FIGURE 2:**
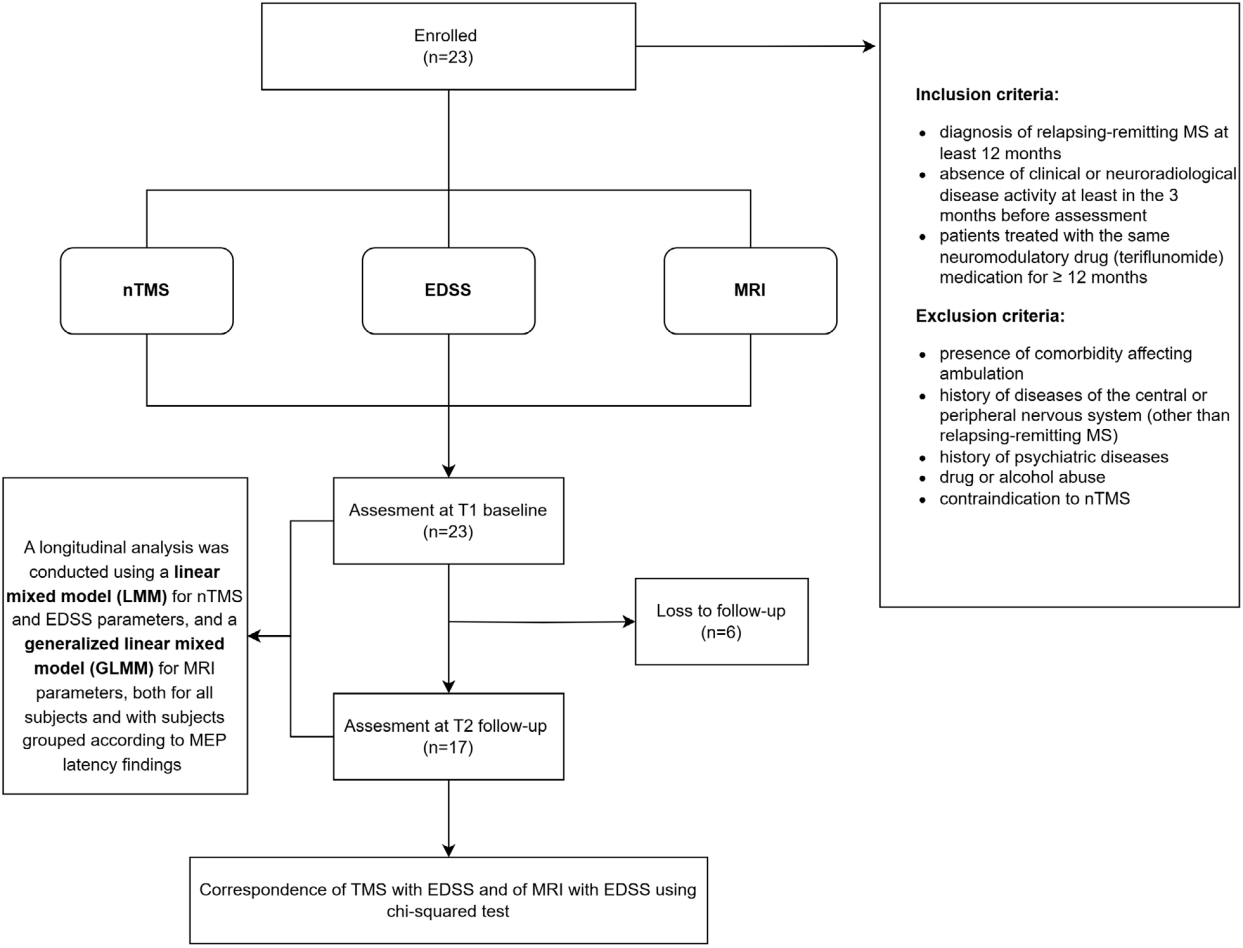
Study design and analysis flowchart. Twenty‐three subjects with relapsing–remitting MS were assessed at baseline (T1) for nTMS, EDSS, and MRI parameters. At the follow‐up (T2), data from 17 subjects were collected. A linear mixed model (LMM) was applied to nTMS and EDSS parameters, and a generalized linear mixed model (GLMM) to MRI parameters, both for all subjects and by grouping the subjects based on their MEP latency findings. Correspondence with EDSS was tested using chi‐squared analysis. EDSS, expanded disability status scale; MEP, motor evoked potential; MRI, magnetic resonance imaging; MS, multiple sclerosis; nTMS, electric‐field navigated transcranial magnetic stimulation.

The level of statistical significance was set to 0.05. Statistical analyses were performed using the statistical programming language R (R Foundation for Statistical Computing, Vienna, Austria) [40]. Figure 2 presents the study design and analysis flowchart.

## 3 Results

Table 1 presents the demographic characteristics at baseline and 2‐year follow‐up. At baseline, the study included 23 patients with MS, 14 women, and 9 men, with a mean age of 41 (SD: 8.89, age range: 24–53) and median EDSS 2.04 (Q1–Q3: 0.5–3.5) (Tables 1 and 2). In the follow‐up, six subjects (26%) refused to participate and the sample consisted of 17 RRMS, 8 women, and 9 men with median EDSS 2 (Q1–Q3: 1–3.5) (Table 1). There was no significant difference in the sex distribution among the participants at baseline and on follow‐up (*p* = 0.297 and *p* = 0.808, Table 1). However, there was a significant difference, both at baseline and at the follow‐up, in the distribution of handedness (*p* < 0.001 and *p* = 0.002), with most of the subjects being right‐handed (91.3% at baseline and 88.24% at follow‐up). Additionally, there were significant differences, both at baseline and at the follow‐up, in the distribution of education (both *p* < 0.001), BMI categories (*p* = 0.003 and *p* = 0.0009), working status (both *p* = 0.005), and smoking (*p* = 0.002 and *p* = 0.011) (Table 1). The median MS disease duration was 7 (Q1–Q3: 6–12) years, and all subjects were treated with teriflunomide for 3.68 ± 1.76 years at the baseline. At follow‐up, two subjects changed immunomodulatory drug (subject No. 1 switched to cladribine and subject No. 19 switched to ocrelizumab), while other RRMS continued with the teriflunomide treatment. Table 2 presents nTMS scores (intensity of stimulation, MEP latency, MEP amplitude) for upper and lower extremity muscles, the EDSS pyramid score for the upper and lower extremities, and psychological and physical status scores (DASS‐21, MSIS‐29) accounting for psychological and physical disability.

**FIGURE 3:**
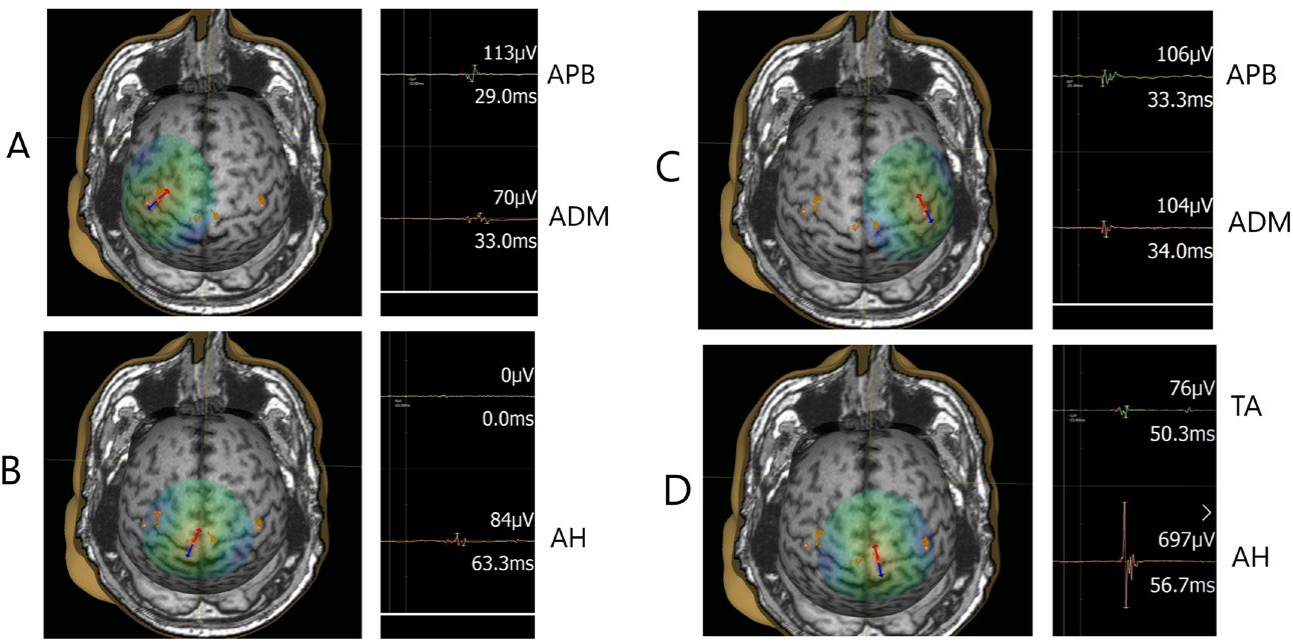
Single subject (No. 3) with altered MEP latency findings for upper and lower extremity muscles at baseline. Left hemisphere stimulation (A, B) and right hemisphere stimulation (C, D) with single trial MEP recordings from upper extremity (APB, ADM) and lower extremity (TA, AH) muscles. ADM, abductor digiti minimi; APB, abductor pollicis brevis; AH, abductor hallucis; TA, tibialis anterior.

**FIGURE 4:**
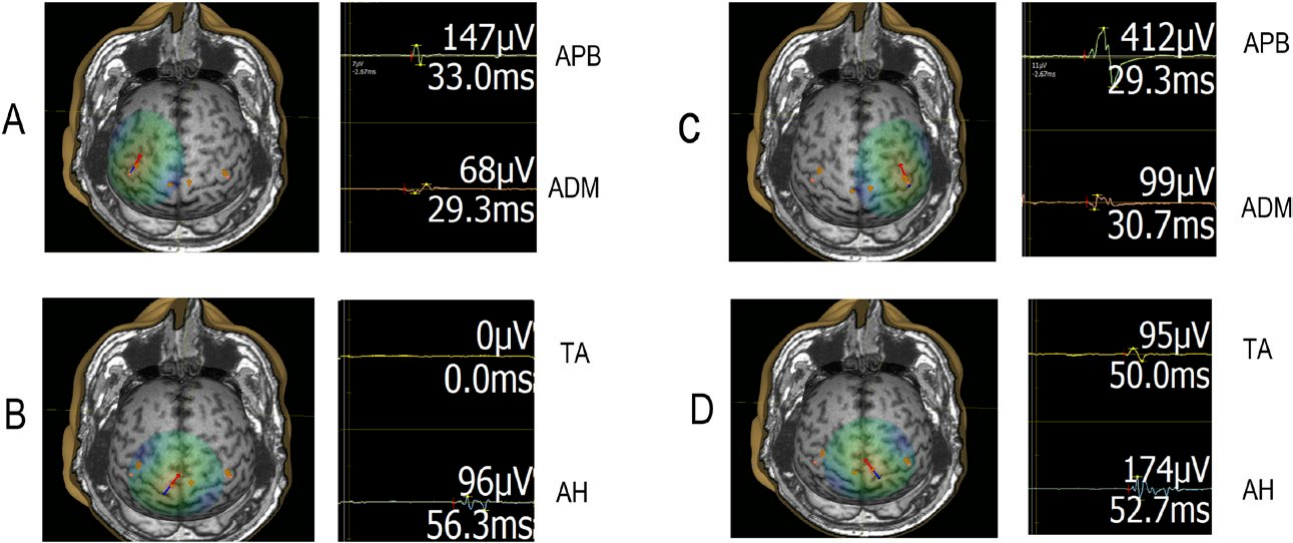
Single subject (No. 3) with altered MEP latency findings for upper and lower extremity muscles at follow‐up. Left hemisphere stimulation (A, B) and right hemisphere stimulation (C, D) with single trial MEP recordings from upper extremity (APB, ADM) and lower extremity (TA, AH) muscles; ADM, abductor digiti minimi; AH, abductor hallucis; APB, abductor pollicis brevis; TA, tibialis anterior.

At baseline, 15 out of 23 subjects had altered MEP latency findings (MEP prolonged or absent) (Table 3) and 8 had non‐altered (normal) MEP latency findings. After 2 years, all patients that had altered MEP latency findings at baseline sustained to have altered MEP latency findings at follow‐up, and no patient from the non‐altered MEP latency group (baseline) showed worsening in MEP latency status at follow‐up. Specifically, six RRMS subjects with non‐altered findings in MEP latency at baseline showed no deterioration in MEP latency findings at the follow‐up, while two subjects with non‐altered findings in MEP latency at baseline withdrew from the study at the follow‐up. Four subjects with altered MEP latency findings at the baseline withdrew from the study at the follow‐up. Summary data for a single subject (No. 3) with altered MEP latency, including EDSS and MRI scores at baseline and follow‐up is presented in Figures 3 and 4; Table 4.

**TABLE 4:** Summary data for a single subject (No. 3) with altered MEP latency findings, including EDSS, and MRI scores for baseline and follow‐up.

	Baseline	Follow‐up
EDSS general	3.5	5
EDSS pyramidal	3	4.5
EDSS right leg	3	4
EDSS left leg	1	3
EDSS right arm	3	3
EDSS left arm	3	2.5
*Right hemisphere nTMS*		
APB		
RMT (%)	37	37
MEP latency (ms)	30	29.7
ADM		
RMT (%)	37	37
MEP latency (ms)	30	28.6
TA		
RMT (%)	80	70
MEP latency (ms)	MEP not elicited	50
AH		
RMT (%)	80	70
MEP latency (ms)	58.1	52.4
*Left hemisphere nTMS*		
APB		
RMT (%)	36	39
MEP latency (ms)	31.7	28.3
ADM		
RMT (%)	42	39
MEP latency (ms)	30.7	27.9
TA		
RMT (%)	79	100
MEP latency (ms)	47	MEP not elicited
AH		
RMT (%)	79	91
MEP latency (ms)	55.3	52.6
*MRI*		
Total number of lesions	14	15
Corticospinal tract	4	4
Corticospinal tract right	2	2
Corticospinal tract left	2	2

*Note:* MEP latency (ms) is an average value.

### 3.1 Linear Mixed Model Results for TMS (MEP) Measures (*All RRMS Subjects*)

#### 3.1.1 Left Hemisphere Stimulation

##### 3.1.1.1 RMT

The results indicated a significant increase in RMT intensity for APB muscle at follow‐up compared to baseline (*β* = 1.53, SE = 0.51, *p* = 0.008), with an increase of 1.53 units over the 2‐year follow‐up period. Additionally, longer MS disease duration was associated with higher RMT intensity values for APB muscle (*β* = 0.99, SE = 0.28, *p* = 0.002). Men exhibited a trend toward lower RMT intensity for the APB muscle compared to women (*β* = −6.31, SE = 3.28, *p* = 0.069) (Supporting Information S1, Table S1.1). Longer MS disease duration was also associated with a significant increase in RMT intensity for ADM muscle (*β* = 0.86, SE = 0.40, *p* = 0.042) (Supporting Information S1, Table S1.4). The results indicated no significant change in RMT intensity for lower extremity TA and AH muscles between baseline and follow‐up assessments. Neither age, sex, nor MS disease duration showed significant effects on RMT intensity for lower extremity muscles (Supporting Information S1).

##### 3.1.1.2 MEP Latency

The results showed that there was no significant change in MEP latency for APB and ADM muscle between baseline and follow‐up assessments (Supporting Information S1). Additionally, neither age, sex, nor MS disease duration significantly affected MEP latency in APB and ADM muscles (Supporting Information S1).

For lower extremity muscles, the results indicated a significant reduction in MEP latency for TA (*β* = −1.61, SE = 0.46, *p* = 0.005, Supporting Information S1, Table S1.8) and AH (*β* = −1.48, SE = 0.57, *p* = 0.022, Supporting Information S1, Table S1.11) muscles at follow‐up compared to baseline with a decrease of 1.61 units in TA and 1.48 units in AH over the 2‐year follow‐up period. Neither age, sex, nor MS disease duration showed significant effects on MEP latency for lower extremity TA and AH muscles (Supporting Information S1).

##### 3.1.1.3 MEP Amplitude

The results indicated that there was no significant change in MEP amplitude for APB, ADM, TA, and AH between baseline and follow‐up assessments. Neither age nor sex had significant effects on MEP amplitude for these muscles (Supporting Information S1). However, longer MS disease duration was associated with changes in MEP amplitude for APB (*β* = 10.57, SE = 4.81, *p* = 0.038) (Supporting Information S1, Table S1.3).

#### 3.1.2 Right Hemisphere Stimulation

##### 3.1.2.1 RMT

The results showed that there was a significant effect of sex, with men having significantly lower RMT intensity for APB (*β* = −11.78, SE = 5.35, *p* = 0.040, Supporting Information S1, Table S2.1) and ADM (*β* = −13.75, SE = 4.97, *p* = 0.013, Supporting Information S1, Table S2.4) compared to women. Additionally, longer MS disease duration was associated with a significant increase in RMT intensity for APB (*β* = 1.01, SE = 0.46, *p* = 0.035). The results indicated no significant change in RMT intensity for lower extremity TA and AH muscles between baseline and follow‐up assessments. Neither age, sex, nor MS disease duration showed significant effects on RMT intensity for lower extremity muscles (Supporting Information S1).

##### 3.1.2.2 MEP Latency

The results showed that there was no significant change in MEP latency for APB and ADM muscles between baseline and follow‐up assessments. Additionally, neither age, sex, nor MS disease duration significantly affected MEP latency in APB and ADM muscles (Supporting Information S1). No significant change in MEP latency for TA and AH muscles between baseline and follow‐up assessments was found. Age, sex, and MS disease duration had no significant effect on MEP latency in TA (Supporting Information S1). However, sex was found to have a significant effect, with men having significantly higher MEP latency in AH compared to women (*β* = 4.35, SE = 1.73, *p* = 0.023).

##### 3.1.2.3 MEP Amplitude

The results indicated no significant change in MEP amplitude for APB, ADM, TA, and AH muscles between baseline and follow‐up assessments. Neither age, sex nor MS disease duration had significant effects on MEP amplitude for these muscles (Supporting Information S1).

### 3.2 Linear Mixed Model Results for EDSS

#### 3.2.1 All RRMS Subjects

The results indicated no significant change in general EDSS, pyramidal EDSS, and pyramidal EDSS scores for each extremity between baseline and follow‐up assessments. Age, sex, and MS disease duration showed no significant effects on EDSS over time in this cohort (Supporting Information S2).

#### 3.2.2 RRMS With Non‐Altered and Altered MEP Findings

The results indicated a significant difference in general EDSS scores at baseline between the MEP latency altered and non‐altered RRMS groups (*β* = −2.06, SE = 0.67, *p* = 0.006, Supporting Information S2, Table S2.1), with the altered MEP latency group having higher EDSS scores. Regarding pyramid EDSS scores, RRMS patients with altered MEP latency findings had higher scores in comparison to RRMS with non‐altered MEP latency findings (*β* = −1.9, SE = 0.57, *p* = 0.002, Supporting Information S2, Table S2.2). Furthermore, the results indicated a significant difference in EDSS pyramid scores for lower extremities between the MEP latency altered and non‐altered RRMS groups, with higher scores in the right (*β* = −1.70, SE = 0.46, *p* = 0.001) and left leg (*β* = −1.50, SE = 0.65, *p* = 0.032) in the altered MEP latency group (Supporting Information S2).

### 3.3 Linear Mixed Model and Generalized Linear Mixed Model Results for Radiological MRI Assessment

#### 3.3.1 All RRMS Subjects

There was no significant change in the total number of lesions between baseline and follow‐up assessments (*β* = 9.73 × 10^−6^, SE = 0.081, *p* = 0.999, Supporting Information S3, Table S1.1). Age also did not significantly affect the total number of lesions (*β* = 1.27 × 10^−2^, SE = 0.023, *p* = 0.996). There was no significant difference in total lesion count between men and women (*β* = 0.089, SE = 0.323, *p* = 0.781). Also, no significant change between baseline and follow‐up was found in the number of lesions respectively for (juxta) cortical, periventricular, infratentorial, spinal cord, corpus callosum, and at CST level (spinal cord—right and left side, CST at primary motor cortex level—right and left side) (Supporting Information S3).

#### 3.3.2 RRMS With Non‐Altered and Altered MEP Findings

There was no significant difference in the total number of lesions between the RRMS with non‐altered and altered MEP findings at baseline (*β* = −0.01, SE = 0.44, *p* = 0.980, Supporting Information S3, Table S2.1). Similarly, no significant change in the total number of lesions over time was observed (*β* = −0.003, SE = 0.943, *p* = 0.972). Age (*β* = −1.82 × 10^−5^, SE = 0.026, *p* = 0.999) and sex (*β* = 0.087, SE = 0.37, *p* = 0.814) were not significant predictors. There was a significant difference in the number of lesions between RRMS patients with non‐altered and altered MEP findings at baseline, with RRMS patients with altered MEP findings having more lesions at all levels, specifically in the corpus callosum region (*β* = 2.38, SE = 1.09, *p* = 0.028) (Supporting Information S3).

### 3.4 Results of Correspondence of TMS and MRI Data With EDSS Scores

#### 3.4.1 All RRMS Subjects

The results of McNemar's test suggest no significant difference in the congruence of TMS scoring with EDSS pyramidal scoring for each extremity between baseline and follow‐up (Supporting Information S4, Table S1.1), indicating correspondence between TMS and EDSS scoring. Also, no significant difference was found in the congruence of MRI scoring with the EDSS pyramidal scoring for each extremity, between baseline and follow‐up, indicating correspondence of MRI and EDSS scoring.

#### 3.4.2 RRMS With Non‐Altered and Altered MEP Findings

No significant difference in the congruence of MRI scoring with EDSS pyramidal scoring for each extremity was found between patients with non‐altered and altered MEP latency at baseline and follow‐up separately, indicating further correspondence of MRI and EDSS scoring (Supporting Information S4). As the MEP latency grouping was directly based on TMS measures, we omitted the analysis of the congruence between TMS scoring and EDSS pyramidal scoring in patients with altered and non‐altered MEP latency, as this would introduce circular reasoning by comparing non‐independent variables and potentially lead to a biased interpretation.

### 3.5 Linear Mixed Model Results for Patient‐Derived Measures (DASS‐21, MSIS‐29)

The LMM results showed no significant effect of time on depression, anxiety, stress, or physical and psychological measures evaluated with DASS‐21, and MSIS‐29 physical and psychologic scores. In all LMM analyses, MS disease duration, age, and sex were not significant predictors (Supporting Information S5).

## 4 Discussion

In the current nTMS study, we examined 23 patients (17 patients in follow‐up) with RRMS longitudinally over 2 years to investigate MEP scores over time in addition to EDSS, MRI lesion count scores, and patient‐derived psychological and physical status investigated by DASS‐21 and MSIS‐29. In parallel to clinical findings, no significant changes were observed in MEP scores, EDSS scores (general, pyramidal), and the total number of MRI lesions after a 2‐year follow‐up. The psychological and physical status of subjects remained stable after 2 years. RRMS subjects with altered MEP latency findings (prolonged MEP latency or absent MEP response) compared to subjects with non‐altered MEP latency findings, had higher EDSS scores in lower extremity muscles as well as a higher number of lesions in the corpus callosum, pointing to the congruent scoring of MRI and nTMS (MEP latency) with EDSS. MS disease duration was associated with higher RMT intensity for upper extremity muscles with men tending to have lower RMT compared to women. Further, MEP latency was found to be shortened in TA and AH muscle at follow‐up compared to baseline for left hemisphere stimulation. These MEP latency findings for lower extremity muscles should be interpreted with caution due to the small sample size with more male subjects represented in the group of subjects with altered MEP latency findings (nine male subjects vs. two women) at follow‐up, and due to cases of absent MEP responses in lower extremity muscles at the maximal stimulator output in this group.

To date, several studies have shown that MEP latency provides congruent information on the function of the CST and is closely related to clinical disability (EDSS) [26, 41] and with lesions and atrophy located in the brain and spinal cord [42]. In the present study we found a significantly greater number of lesions in the corpus callosum in RRMS subjects with altered MEP findings compared to RRMS with non‐altered MEP findings (Supporting Information S3). The findings of our study point out that the evaluation of the CST lesions at three specific levels, which was performed in addition to the standard McDonald criteria, was less informative in explaining MEP findings. According to Morozumi et al. [42], various combinations of brain and cervical spinal MRI measures explain EDSS and the relevance of MRI variables varies across specific EDSS scores (i.e., in lower EDSS scores combined brain and cervical spinal cord measures have similar importance, and in higher EDSS scores, such as EDSS 6.0, the cervical spinal cord damage is more relevant). Therefore, previous studies suggest that brain MRI measures are more relevant in explaining mild disability [43], which may also apply to the present study's MRI findings and our RRMS sample, which had a mild EDSS score (2.5 at baseline and 2.0 at follow‐up).

Along with similar findings of our nTMS study, Fuhr and Kappos prospectively evaluated 30 MS patients (RRMS or secondary‐progressive—SPMS) longitudinally over 2 years and demonstrated a robust correlation between EDSS score and MEP latency [44]. Several retrospective and prospective studies have reported that the extent of change in the composite/multimodal EP scores (combination of two or all modalities MEP, VEP, SEP) are significantly related to future EDSS scores over two up to 20 years in cohorts with clinically isolated syndrome (CIS) [45], RRMS [46, 47], primary progressive MS (PPMS) [27], and in mix cohorts of RRMS, SPMS and PPMS [10, 13, 48]. The combination of VEP, MEP from upper and lower extremity muscles and tibial SEP has proved to have the highest prognostic correlation to EDSS baseline (range 2.0–6.5) in PPMS over 3 years [30], while VEP, SEP, and MEP from lower extremity muscles showed highest prognostic correlation to EDSS in RRMS [26, 48].

Considering the single EP modalities and the fact that the functional pyramidal EDSS system score was shown to be the most frequently associated with sustained disability progression in RRMS, MEPs have been shown to correlate with measures of motor performance, to improve under treatment and to predict disability worsening [49, 50, 51, 52, 53]. Therefore, MEPs may represent a supreme candidate marker for objective assessment and monitoring of motor disability and testing remyelination strategies in MS [23, 35, 54]. In the present nTMS study, MEP was used as a single EP modality for longitudinal monitoring of the CST integrity for upper and lower limbs in RRMS and was found to be reliable concerning the advantages of the nTMS technology. The advantage of the present study is that the electric‐field nTMS system allows inspection of the primary motor cortical representation for the upper and lower extremity muscles in individual subjects by repeating the same target locations at the follow‐up used in the baseline [17, 41]. Nonetheless, to our knowledge, the current study comprises the first cohort of patients with RRMS longitudinally monitored with nTMS assessment by inspecting MEP from four limbs (and four muscles, two muscles per limb). Recently Bardel et al. [55] also used nTMS and single MEP modality to investigate motor function in 68 MS subjects (RRMS, PPMS, and SPMS) by inspecting cortical motor maps in addition to MEP scores (latency, amplitude) in hand (first dorsal interosseus, FDI) and lower extremity muscles (TA), opening the perspectives for the use of nTMS in mapping the CST integrity in clinical settings. Ongoing clinical recommendations for MEP use in MS refer to the application of a magnetic stimulator connected to a standard EMG unit [18], and less to line navigated TMS and electric‐field navigated TMS implementations [16, 17, 19, 55] that could provide more precision in targeting and visualization of the primary motor cortices for single muscle representation and repeating positive hot spots over longitudinal follow‐up.

Our study has several limitations that warrant further discussion. This is a longitudinal, single‐center cohort with a small RRMS with overall mild disability. The baseline general EDSS 2.04 (EDSS 1 pyramidal score for the right and left legs) after the 2‐year follow‐up remained comparable (general EDSS 2 and EDSS 1 pyramidal score for the right leg and EDSS 1.5 for the left leg). Therefore, 2‐year nTMS monitoring applied in our study might not be the optimal time window for inspecting MEP score changes that might be supported by the findings of Zurawski et al. [56] indicating that the differences in the median time to EDSS = 2 and EDSS = 2.5 are of 4.9 years. Zurawski et al. [56] also showed that for EDSS between 2.5 and 3, there is a strong association for functional pyramidal, cerebellar, bowel‐bladder, and mental scores. In the present study, general and pyramidal EDSS scores were obtained with MEP scoring since nTMS mapping of the CST was performed for each limb separately to yield better correspondence of MEP scoring and EDSS pyramidal evaluation. In comparison to Bardel et al. [55] evaluating general EDSS, in our study functional pyramidal EDSS was analyzed for each limb respectively in addition to the general EDSS, providing a more detailed association with MEP evaluation. Further, in the present study, we applied an RMT of 100% to compare baseline and follow‐up MEPs from upper and lower extremity muscles. This approach was chosen because, in some RRMS cases, MEPs from lower extremity muscles could only be elicited at maximal stimulator output, often with lower peak‐to‐peak amplitude. While we acknowledge that MEP peak‐to‐peak amplitude variability could be optimized using 120% RMT intensity across individuals, the baseline measurement did not include M1 mapping to identify the hotspot for upper and lower extremity muscles at RMT 120%. Consequently, the variability of MEP peak‐to‐peak amplitude could not be fully assessed in our RRMS sample.

Furthermore, regarding the psychological and physical impact of the MS disease measured with DASS‐21 and MSIS‐29, the limiting factor might be that these scales measure mostly psychological status, depression, stress, and anxiety in the last one to 2 weeks, which might not be sensitive measures for nTMS longitudinal MEP monitoring of MS patients. Regarding the MRI measure, even though the poor association between conventional MRI measures of tissue damage with EDSS has been reported previously [8], the lesion count in the present study might also not be the single optimal measure for monitoring purposes, and more advanced quantitative MRI measures would be recommended [4, 42, 57, 58]. An example is the multiparametric structural and functional MRI study of Cordani et al. [59], which was conducted on a large sample of MS and control subjects investigating hand motor performance with the Nine‐Hole Peg test. The study findings indicated structural and functional abnormalities of regions involved in motor functions (decreased corticospinal fractional anisotropy) [59].

Lastly, we could not prevent the loss of six RRMS subjects (26%) at follow‐up, as participation was voluntary. However, this attrition may have influenced the study results, particularly the observed shortening of MEP latency in the lower extremity muscles of the right leg at follow‐up compared to baseline during left M1 stimulation. These findings were unexpected and may be attributed to the reduced sample size at follow‐up and the absence of detectable MEP responses in the legs in some cases.

## 5 Conclusion

The results highlight the value of nTMS assessment in the longitudinal evaluation of CST integrity by inspecting MEP scores from four limbs in addition to MRI, EDSS, and psychological evaluation. The findings from the present study point to the practical contribution of nTMS mapping of the CST integrity to EDSS evaluation. Overall, future longitudinal nTMS studies might take advantage of the strengths of the present study (representative number of target muscles for MEP recording—APB, ADM, TA, AH; evaluation of functional pyramidal EDSS score for each limb in addition to general EDSS score) and by taking into account the reported findings on the optimal time scale for disability accumulation by general EDSS and functional EDSS scores [56], additional nTMS measures (i.e., the size of cortical maps) [56] and including more advanced MRI protocols [42, 58], information on the previous rehabilitation treatments, and including sensitive measures of physical and cognitive status (i.e., the Nine‐Hole Peg test, 25‐Foot Walk, SDMT, etc.) [59, 60].

## Author Contributions

**Antonia Bralić:** involved in study conception, design, patient recruitment, patient assessments, and drafting and revisions of the manuscript. **Sanda Pavelin:** involved in study conception, design, patient recruitment, patient assessments, and drafting and revisions of the manuscript. **Nikolina Pleić:** statistical analysis, and drafting and revisions of manuscript. **Joško Šoda:** involved in study conception, methodological development of software for MEP evaluation, and manuscript drafting and revisions. **Krešimir Dolić:** involved in study conception, design, patient recruitment, patient assessments, and drafting and revisions of the manuscript. **Anita Markotić:** involved in study conception, design, and manuscript drafting and revisions. **Ana Ćurković Katić:** involved in study conception, design, patient recruitment, patient assessments, and drafting and revisions of the manuscript. **Angela Mastelić:** involved in study conception, design, and manuscript drafting and revisions. **Nikolina Režić Mužinić:** involved in study conception, design, and manuscript drafting and revisions. **Jasna Duranović:** statistical analysis, and drafting and revisions of manuscript. **Zlatko Kljajić:** involved in study conception, drafting, and revising the manuscript. **Ivona Stipica Safić:** involved in study conception, drafting, and revising the manuscript. **Zoran Đogaš:** involved in study conception, drafting, and revising the manuscript. **Maja Rogić Vidaković:** involved in study conception, design, obtaining ethical approval, patient recruitment, patient assessments, MEP data analysis, and drafting and revisions of the manuscript.

## Ethics Statement

The study procedure was approved by the Ethical Committee board of School of Medicine, University of Split (Class: 003‐08/21‐03/0003, No: 2181‐198‐03‐04‐21‐0039) (February 21, 2021), second annex (Class: 003‐081/22‐03/0003, No: 2181‐198‐03‐04‐22‐0021) (March 29, 2022), and third annex (Class: 003‐08/23‐03/0015; No: 2181‐198‐03‐04‐23‐0075) (September 27, 2023). The study procedure was approved by the Ethical Committee board of University Hospital of Split research (Class: 500‐03/20‐01/06, No: 2181‐147‐01‐06/M.S.‐20‐02) (June 27, 2021), second annex (500‐03/20‐01/06, No: 2181‐147‐01‐06/Lj.Z.‐23‐04) (September 20, 2023), and third annex (Class: 500‐03/20‐01/06, No: 2181‐147/01/06/LJ.Z.‐23‐06). The study is in accordance with the Declaration of Helsinki.

## Consent

Each participant gave his/her consent to the study by signing the informed consent.

## Conflicts of Interest

The authors declare no conflicts of interest.

## Supporting information


Supporting Information S1.

Supporting Information S2.

Supporting Information S3.

Supporting Information S4.

Supporting Information S5.

## Data Availability

The data that support the findings of this study are available from the corresponding author upon reasonable request.
