# Supplementary material for: Correspondence of MRI and nTMS With EDSS in Multiple Sclerosis: Longitudinal Follow‐Up Study
Source: Ann Clin Transl Neurol. 2025 Apr 17;12(6):1240–55. doi: 10.1002/acn3.70041 (PMC12172135; doi:10.1002/acn3.70041)
Supplement: Supplementary file 1 — Supporting Information S1. [file ACN3-12-1240-s004.docx]

**Supplementary information S1**

**Detailed linear mixed model (LMM) results for TMS (MEP) parameters**

All relapsing-remitting multiple sclerosis (RRMS) participants

**Table of Contents**

[1. TMS left hemisphere stimulation 2](#_Toc181200543)

[1.1. APB RMT (%) 2](#_Toc181200544)

[1.2. APB MEP latency 3](#_Toc181200545)

[1.3. APB MEP amplitude 4](#_Toc181200546)

[1.4. ADM RMT (%) 5](#_Toc181200547)

[1.5. ADM MEP latency 6](#_Toc181200548)

[1.6. ADM MEP amplitude 7](#_Toc181200549)

[1.7. TA RMT (%) 8](#_Toc181200550)

[1.8. TA MEP latency 9](#_Toc181200551)

[1.9. TA MEP amplitude 10](#_Toc181200552)

[1.10. AH RMT (%) 11](#_Toc181200553)

[1.11. AH MEP latency 12](#_Toc181200554)

[1.12. AH MEP amplitude 13](#_Toc181200555)

[2. TMS right hemisphere stimulation 14](#_Toc181200556)

[2.1. APB RMT (%) 14](#_Toc181200557)

[2.2. APB MEP latency 15](#_Toc181200558)

[2.3. APB MEP amplitude 16](#_Toc181200559)

[2.4. ADM RMT (%) 17](#_Toc181200560)

[2.5. ADM MEP latency 18](#_Toc181200561)

[2.6. ADM MEP amplitude 19](#_Toc181200562)

[2.7. TA RMT (%) 20](#_Toc181200563)

[2.8. TA MEP latency 21](#_Toc181200564)

[2.9. TA MEP amplitude 22](#_Toc181200565)

[2.10. AH RMT (%) 23](#_Toc181200566)

[2.11. AH MEP latency 24](#_Toc181200567)

[2.12. AH MEP amplitude 25](#_Toc181200568)

A linear mixed-effects model was employed to evaluate the impact of time, age, sex, and disease duration on MRI parameters in individuals with multiple sclerosis. The models included Time (Baseline vs. Follow-up) as a fixed effect, with age, sex (coded as 1 = female, 2 = male), and MS disease duration as covariates. A random intercept for each participant was included to account for the correlation of repeated measures within the same individuals.

# 1. TMS left hemisphere stimulation

## 1.1. APB RMT (%)

Table S1.1. Results of the linear mixed model longitudinal analysis for APB RMT (%)

**
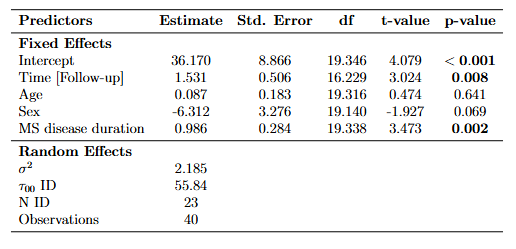
**

τ₀₀ (Tau), the variance of the random intercepts for the grouping factor (ID) representing the variability in the baseline levels between groups; σ², residual variance representing the within-group variability (i.e., the variability not explained by the grouping factor). Significant p-values are marked in bold.

The results indicated a significant increase in APB RMT at follow-up compared to baseline (β = 1.53, SE = 0.51, p = 0.008), suggesting that motor threshold levels rise over time in this cohort, with an increase of 1.53 units in APB RMT over the 2-year follow-up period. Additionally, longer MS disease duration was associated with higher APB RMT values (β = 0.99, SE = 0.28, p = 0.002), indicating that for each additional year of disease duration, APB RMT increases by 0.99 units, suggesting that disease progression contributes to increased motor excitability. The effect of sex showed a trend towards significance, with men tending to have lower APB RMT compared to women (β = -6.31, SE = 3.27, p = 0.069), although this was not statistically significant. Age did not significantly influence APB RMT (β = 0.09, SE = 0.18, p = 0.641).

The random effect for participants, with a variance of 55.838, suggests a large amount of inter-individual variability in baseline APB RMT values. In contrast, the residual variance (σ^2^ = 2.185) is smaller, indicating smaller within-individual variability across time points.

## 1.2. APB MEP latency

Table S1.2. Results of the linear mixed model longitudinal analysis for APB MEP latency

**
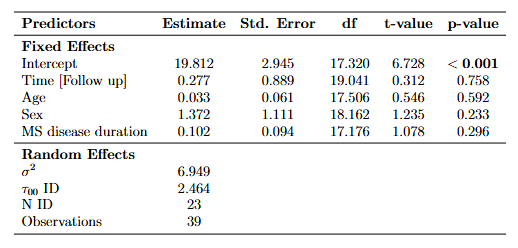
**

τ₀₀ (Tau), the variance of the random intercepts for the grouping factor (ID) representing the variability in the baseline levels between groups; σ², residual variance representing the within-group variability (i.e., the variability not explained by the grouping factor). Significant p-values are marked in bold.

The results showed that there was no significant change in APB MEP latency between baseline and follow-up assessments (β = 0.28, SE = 0.89, p = 0.758), indicating that time alone did not significantly influence motor-evoked potential latency in this cohort. Additionally, neither age (β = 0.03, SE = 0.06, p = 0.592) nor MS disease duration (β = 0.10, SE = 0.09, p = 0.296) had a significant effect on APB MEP latency, suggesting that these factors do not substantially contribute to variability in latency measures in this population. While men had a higher latency compared to women (β = 1.37, SE = 1.11, p = 0.233), this difference was not statistically significant.

The random effect for participants, with a variance of 2.464, suggests a small amount of inter-individual variability in baseline APB MEP latency values. In contrast, the residual variance (σ^2^ = 6.949) is larger, indicating larger within-individual variability across time points.

## 1.3. APB MEP amplitude

Table S1.3. Results of the linear mixed model longitudinal analysis for APB MEP amplitude

**
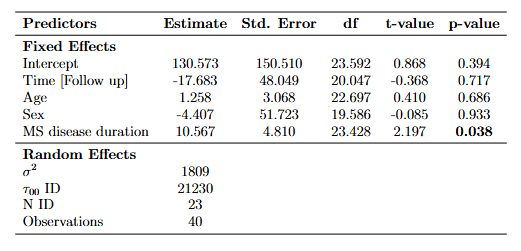
**

τ₀₀ (Tau), the variance of the random intercepts for the grouping factor (ID) representing the variability in the baseline levels between groups; σ², residual variance representing the within-group variability (i.e., the variability not explained by the grouping factor). Significant p-values are marked in bold.

The results indicated that there was no significant change in APB MEP amplitude between baseline and follow-up assessments (β = -17.683, SE = 48.049, p = 0.717), suggesting that time alone did not significantly influence MEP amplitude in this cohort. Neither age (β = 1.258, SE = 3.068, p = 0.686) nor sex (β = -4.407, SE = 51.723, p = 0.933) had significant effects on APB MEP amplitude. However, longer MS disease duration was associated with a significant increase in APB MEP amplitude (β = 10.567, SE = 4.810, p = 0.038), indicating that for each additional year of disease duration, APB MEP amplitude increases by 10.567 units, suggesting that disease progression may lead to higher motor evoked potential amplitudes.

The random effect for participants, with a variance of 1809, suggests inter-individual variability in baseline APB MEP amplitude values. In contrast, the residual variance (σ^2^ = 21230) is substantially larger, indicating substantial within-individual variability across time points.

## 1.4. ADM RMT (%)

Table S1.4. Results of the linear mixed model longitudinal analysis for ADM RMT (%)

**
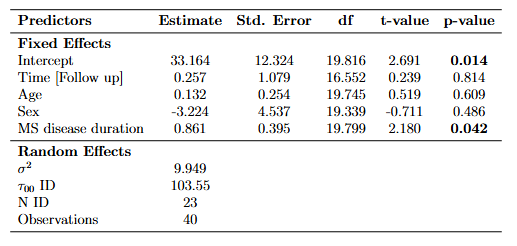
**

τ₀₀ (Tau), the variance of the random intercepts for the grouping factor (ID) representing the variability in the baseline levels between groups; σ², residual variance representing the within-group variability (i.e., the variability not explained by the grouping factor). Significant p-values are marked in bold.

The analysis revealed that there was no significant change in ADM RMT between baseline and follow-up assessments (β = 0.257, SE = 1.079, p = 0.814), indicating that time alone did not significantly influence motor threshold in this cohort. Neither age (β = 0.132, SE = 0.254, p = 0.609) nor sex (β = -3.224, SE = 4.537, p = 0.486) showed significant effects on ADM RMT, suggesting these factors do not substantially impact motor threshold variability. However, longer MS disease duration was associated with a significant increase in ADM RMT (β = 0.861, SE = 0.395, p = 0.042), indicating that for each additional year of disease duration, ADM RMT increases by 0.861 units, suggesting that disease progression may contribute to higher motor thresholds.

The random effect for participants, with a variance of 103.55, suggests a substantial amount of inter-individual variability in baseline ADM RMT values. In contrast, the residual variance (σ^2^ = 9.949) is smaller, indicating smaller within-individual variability across time points.

## 1.5. ADM MEP latency

Table S1.5. Results of the linear mixed model longitudinal analysis for ADM MEP latency

**
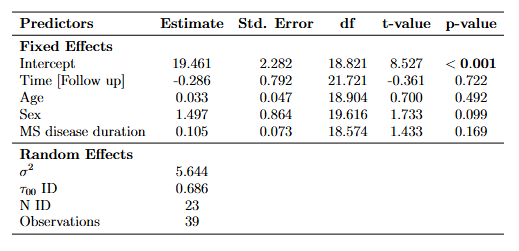
**

τ₀₀ (Tau), the variance of the random intercepts for the grouping factor (ID) representing the variability in the baseline levels between groups; σ², residual variance representing the within-group variability (i.e., the variability not explained by the grouping factor). Significant p-values are marked in bold.

The results showed that there was no significant change in ADM MEP latency between baseline and follow-up assessments (β = -0.286, SE = 0.792, p = 0.722), indicating that time alone did not significantly affect motor-evoked potential latency in this cohort. Neither age (β = 0.033, SE = 0.047, p = 0.492) nor MS disease duration (β = 0.105, SE = 0.073, p = 0.169) had significant effects on ADM MEP latency. The effect of sex, while not reaching statistical significance, showed a trend where men had a slightly longer latency compared to women (β = 1.497, SE = 0.864, p = 0.099).

The random effect for participants, with a variance of 0.686, suggests a small amount of inter-individual variability in baseline MEP latency values. In contrast, the residual variance (σ^2^ = 5.644) is larger, indicating substantial within-individual variability across time points.

## 1.6. ADM MEP amplitude

Table S1.6. Results of the linear mixed model longitudinal analysis for ADM MEP amplitude

**
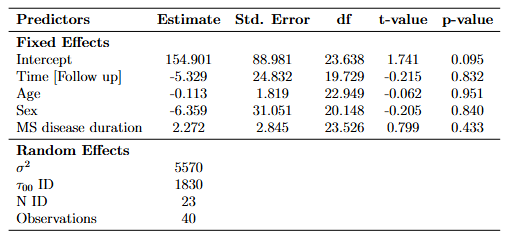
**

τ₀₀ (Tau), the variance of the random intercepts for the grouping factor (ID) representing the variability in the baseline levels between groups; σ², residual variance representing the within-group variability (i.e., the variability not explained by the grouping factor). Significant p-values are marked in bold.

The results indicated that there was no significant change in ADM MEP amplitude between baseline and follow-up assessments (β = -5.329, SE = 24.832, p = 0.832), suggesting that time alone does not significantly influence MEP amplitude in this cohort. Neither age (β = -0.113, SE = 1.819, p = 0.951) nor MS disease duration (β = 2.272, SE = 2.845, p = 0.433) had significant effects on ADM MEP amplitude. The effect of sex, although not statistically significant, showed a slight trend where men had a lower amplitude compared to women (β = -6.359, SE = 31.051, p = 0.840).

The random effect for participants, with a variance of 1830, indicates substantial inter-individual variability in baseline ADM MEP amplitude values. In contrast, the residual variance (σ^2^ = 5570) is much larger, indicating even greater within-individual variability across time points.

## 1.7. TA RMT (%)

Table S1.7. Results of the linear mixed model longitudinal analysis for TA RMT (%)


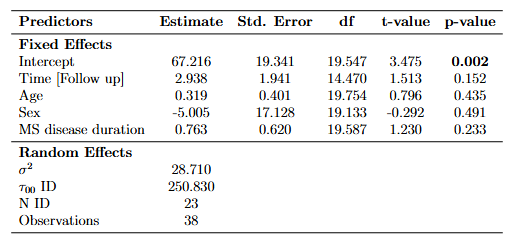


τ₀₀ (Tau), the variance of the random intercepts for the grouping factor (ID) representing the variability in the baseline levels between groups; σ², residual variance representing the within-group variability (i.e., the variability not explained by the grouping factor). Significant p-values are marked in bold.

The results indicated that there was no significant change in TA RMT between baseline and follow-up assessments (β = 2.94, SE = 1.94, p = 0.152), suggesting that time alone does not significantly influence motor threshold in this cohort. Neither age (β = 0.32, SE = 0.40, p = 0.435) nor sex (β = -5.01, SE = 7.13, p = 0.491) showed significant effects on TA RMT, indicating that these factors do not substantially impact motor threshold variability in this sample. MS disease duration also did not have a significant effect on TA RMT (β = 0.76, SE = 0.62, p = 0.233).

The random effect for participants, with a variance of 250.83, indicates substantial inter-individual variability in baseline motor threshold values. The residual standard deviation (σ^2^ = 28.71) suggests smaller within-individual variability across time points.

## 1.8. TA MEP latency

Table S1.8. Results of the linear mixed model longitudinal analysis for TA MEP latency


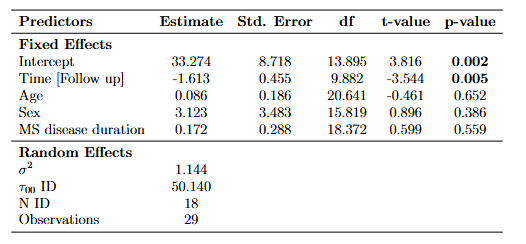


τ₀₀ (Tau), the variance of the random intercepts for the grouping factor (ID) representing the variability in the baseline levels between groups; σ², residual variance representing the within-group variability (i.e., the variability not explained by the grouping factor). Significant p-values are marked in bold.

The results indicated a significant reduction in TA MEP latency at follow-up compared to baseline (β = -1.613, SE = 0.46, p = 0.005), suggesting a decrease in latency over time in this cohort, with a decrease of 1.613 units in TA MEP latency over the 2-year follow-up period. However, neither age (β = -0.086, SE = 0.19, p = 0.652) nor MS disease duration (β = 0.172, SE = 0.29, p = 0.559) significantly influenced TA MEP latency. The effect of sex was also not significant, indicating no meaningful difference in latency between males and females (β = 3.12, SE = 3.49, p = 0.386).

The random effect for participants, with a variance of 50.14, suggests a large amount of inter-individual variability in baseline TA MEP latency values. In contrast, the residual variance (σ^2^ = 1.144) is smaller, indicating smaller within-individual variability across time points.

## 1.9. TA MEP amplitude

Table S1.9. Results of the linear mixed model longitudinal analysis for TA MEP amplitude


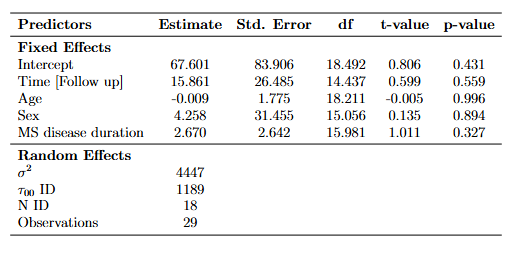


τ₀₀ (Tau), the variance of the random intercepts for the grouping factor (ID) representing the variability in the baseline levels between groups; σ², residual variance representing the within-group variability (i.e., the variability not explained by the grouping factor). Significant p-values are marked in bold.

The results indicated that there was no significant change in TA MEP amplitude between baseline and follow-up assessments (β = 15.86, SE = 24.49, p = 0.559), suggesting that time alone does not significantly influence motor evoked potential amplitude in this cohort. Similarly, neither age (β = -0.009, SE = 1.77, p = 0.996) nor MS disease duration (β = 2.67, SE = 2.64, p = 0.327) showed significant effects on TA MEP amplitude. The effect of sex was also not significant, indicating no meaningful difference in amplitude between males and females (β = 4.26, SE = 31.46, p = 0.894).

The random effect for participants, with a variance of 1189, indicates substantial inter-individual variability in baseline TA MEP amplitude values. In contrast, the residual variance (σ^2^ = 4447) is much larger, indicating even greater within-individual variability across time points.

## 1.10. AH RMT (%)

Table S1.10. Results of the linear mixed model longitudinal analysis for AH RMT (%)


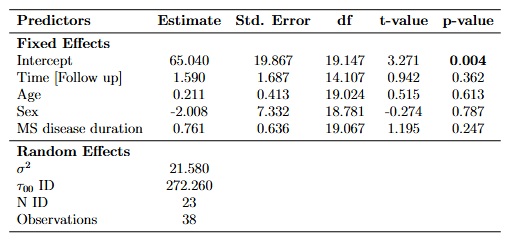


τ₀₀ (Tau), the variance of the random intercepts for the grouping factor (ID) representing the variability in the baseline levels between groups; σ², residual variance representing the within-group variability (i.e., the variability not explained by the grouping factor). Significant p-values are marked in bold.

The results showed no significant change in AH RMT between baseline and follow-up assessments (β = 1.59, SE = 1.69, p = 0.362), indicating that time alone did not significantly influence motor threshold in this cohort. Neither age (β = 0.21, SE = 0.41, p = 0.613) nor MS disease duration (β = 0.76, SE = 0.64, p = 0.247) had significant effects on AH RMT. The effect of sex, although not statistically significant, suggested a trend where males had slightly lower motor thresholds compared to females (β = -2.01, SE = 7.33, p = 0.787).

The random effect for participants, with a variance of 272.26, indicates substantial inter-individual variability in baseline AH RMT values. The residual standard deviation (σ^2^ = 21.58) suggests smaller within-individual variability across time points.

## 1.11. AH MEP latency

Table S1.11. Results of the linear mixed model longitudinal analysis for AH MEP latency


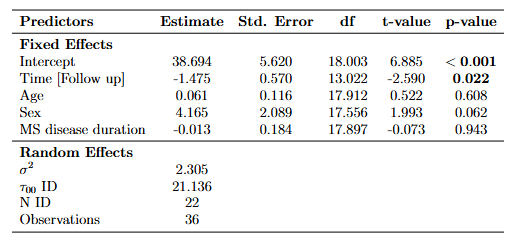


τ₀₀ (Tau), the variance of the random intercepts for the grouping factor (ID) representing the variability in the baseline levels between groups; σ², residual variance representing the within-group variability (i.e., the variability not explained by the grouping factor). Significant p-values are marked in bold.

The results indicated a significant reduction in AH MEP latency at follow-up compared to baseline (β = -1.48, SE = 0.57, p = 0.022), suggesting that, on average, AH MEP latency decreased by 1.48 units over the follow-up period, indicating a decrease in latency over time in this cohort. However, neither age (β = 0.06, SE = 0.12, p = 0.608) nor MS disease duration (β = -0.01, SE = 0.18, p = 0.943) had significant effects on AH MEP latency. The effect of sex was also not significant, indicating no meaningful difference in latency between males and females (β = 4.17, SE = 2.09, p = 0.062).

The random effect for participants, with a variance of 21.136, suggests a moderate amount of inter-individual variability in baseline AH MEP latency values. In contrast, the residual variance (σ^2^ = 2.305) is smaller, indicating smaller within-individual variability across time points.

## 1.12. AH MEP amplitude

Table S1.12. Results of the linear mixed model longitudinal analysis for AH MEP amplitude


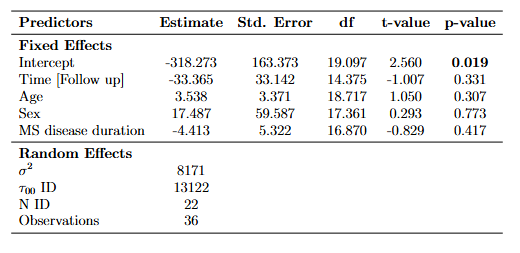


τ₀₀ (Tau), the variance of the random intercepts for the grouping factor (ID) representing the variability in the baseline levels between groups; σ², residual variance representing the within-group variability (i.e., the variability not explained by the grouping factor). Significant p-values are marked in bold.

The results indicated that there was no significant change in AH MEP amplitude between baseline and follow-up assessments (β = -33.37, SE = 33.14, p = 0.331), suggesting that time alone does not significantly influence motor evoked potential amplitude in this cohort. Similarly, neither age (β = -3.54, SE = 3.37, p = 0.3073) nor MS disease duration (β = -4.41, SE = 5.32, p = 0.4174) showed significant effects on AH MEP amplitude. The effect of sex was also not significant, indicating no meaningful difference in amplitude between males and females (β = -17.49, SE = 59.59, p = 0.773).

The random effect for participants, with a variance of 13122, indicates substantial inter-individual variability in baseline AH MEP amplitude values. In contrast, the residual variance (σ² = 8171) is smaller, indicating smaller within-individual variability across time points.

# 2. TMS right hemisphere stimulation

## 2.1. APB RMT (%)

Table S2.1. Results of the linear mixed model longitudinal analysis for APB RMT (%)


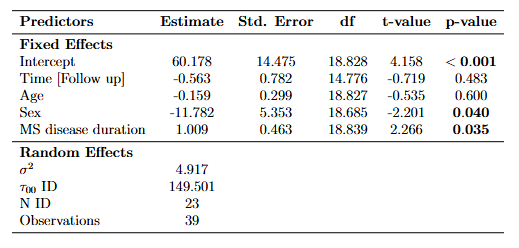


τ₀₀ (Tau), the variance of the random intercepts for the grouping factor (ID) representing the variability in the baseline levels between groups; σ², residual variance representing the within-group variability (i.e., the variability not explained by the grouping factor). Significant p-values are marked in bold.

The results indicated that there was no significant change in APB RMT between baseline and follow-up assessments (β = -0.563, SE = 0.783, p = 0.483), suggesting that time alone does not significantly influence motor threshold in this cohort. Age also did not have a significant effect on APB RMT (β = -0.160, SE = 0.299, p = 0.599), indicating that changes in age are not associated with motor threshold variability in this sample. The effect of sex was significant, with males having significantly lower APB RMT compared to females (β = -11.782, SE = 5.353, p = 0.041), suggesting a lower motor threshold in males. Additionally, longer MS disease duration was associated with a significant increase in APB RMT (β = 1.051, SE = 0.464, p = 0.035), indicating that for each additional year of disease duration, motor thresholds increase by 1.05 units, suggesting that disease progression may contribute to higher motor thresholds.

The random effect for participants, with a variance of 149.5, indicates substantial inter-individual variability in baseline APB RMT values. In contrast, the residual variance (σ² = 4.92) suggests small within-individual variability across time points.

## 2.2. APB MEP latency

Table S2.2. Results of the linear mixed model longitudinal analysis for APB MEP latency

**
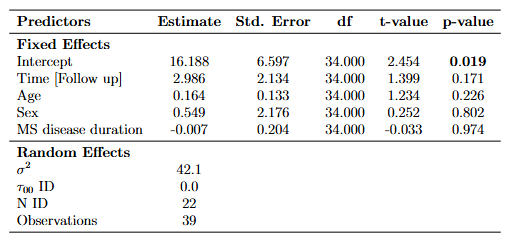
**

τ₀₀ (Tau), the variance of the random intercepts for the grouping factor (ID) representing the variability in the baseline levels between groups; σ², residual variance representing the within-group variability (i.e., the variability not explained by the grouping factor). Significant p-values are marked in bold.

The results indicated that there was no significant change in APB MEP latency between baseline and follow-up assessments (β = 2.99, SE = 2.13, p = 0.171), suggesting that time alone does not significantly influence MEP latency in this cohort. Age also did not have a significant effect on APB MEP latency (β = 0.16, SE = 0.13, p = 0.226), indicating that changes in age are not associated with MEP latency variability in this sample. Neither sex (β = 0.55, SE = 2.18, p = 0.802) nor MS disease duration (β = -0.007, SE = 0.20, p = 0.974) had significant effects on APB MEP latency, suggesting that these factors do not contribute to changes in MEP latency.

The random effect for participants showed a variance of 0, indicating no inter-individual variability in baseline APB MEP latency values within this model. The residual variance (σ² = 42.1) reflects the within-individual variability across time points, which is the primary source of variability in this model.

## 2.3. APB MEP amplitude

Table S2.3. Results of the linear mixed model longitudinal analysis for APB MEP amplitude


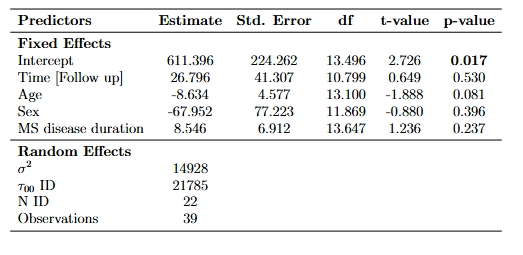


τ₀₀ (Tau), the variance of the random intercepts for the grouping factor (ID) representing the variability in the baseline levels between groups; σ², residual variance representing the within-group variability (i.e., the variability not explained by the grouping factor). Significant p-values are marked in bold.

The results indicated no significant change in APB MEP amplitude between baseline and follow-up assessments (β = 26.80, SE = 41.31, p = 0.530), suggesting that time alone does not significantly influence motor evoked potential amplitude in this cohort. Age showed a non-significant trend towards an inverse relationship with APB MEP amplitude (β = -8.64, SE = 4.58, p = 0.081), indicating that older age may be associated with a slight decrease in amplitude, although this effect did not reach statistical significance. Neither sex (β = -67.95, SE = 77.22, p = 0.396) nor MS disease duration (β = 8.55, SE = 6.91, p = 0.237) had significant effects on APB MEP amplitude, suggesting that these factors do not substantially contribute to changes in motor evoked potential amplitude.

The random effect for participants, with a variance of 21785, indicates substantial inter-individual variability in baseline APB MEP amplitude values. The residual variance (σ² = 14928) suggests additional smaller within-individual variability across time points.

## 2.4. ADM RMT (%)

Table S2.4. Results of the linear mixed model longitudinal analysis for ADM RMT (%)

**
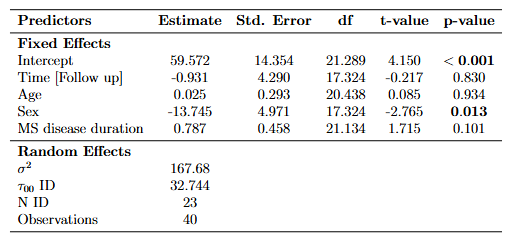
**

τ₀₀ (Tau), the variance of the random intercepts for the grouping factor (ID) representing the variability in the baseline levels between groups; σ², residual variance representing the within-group variability (i.e., the variability not explained by the grouping factor). Significant p-values are marked in bold.

The results indicated no significant change in ADM RMT between baseline and follow-up assessments (β = -0.93, SE = 4.29, p = 0.831), suggesting that time alone does not significantly influence motor threshold in this cohort. Age did not have a significant effect on ADM RMT (β = 0.025, SE = 0.29, p = 0.933), indicating that changes in age are not associated with motor threshold variability in this sample. The effect of sex was significant, with males having significantly lower ADM RMT compared to females (β = -13.74, SE = 4.97, p = 0.013), suggesting a lower motor threshold in males. MS disease duration did not have a significant effect on ADM RMT (β = 0.79, SE = 0.46, p = 0.101), indicating that disease duration is not associated with changes in motor threshold in this sample.

The random effect for participants, with a variance of 32.74, indicates moderate inter-individual variability in baseline ADM RMT values. The residual variance (σ² = 167.68) suggests substantial within-individual variability across time points.

## 2.5. ADM MEP latency

Table S2.5. Results of the linear mixed model longitudinal analysis for ADM MEP latency


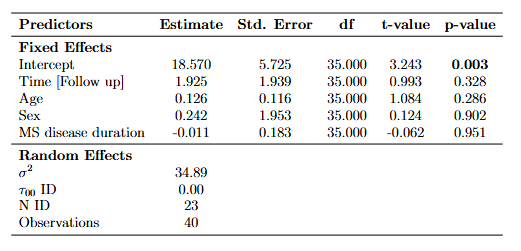


τ₀₀ (Tau), the variance of the random intercepts for the grouping factor (ID) representing the variability in the baseline levels between groups; σ², residual variance representing the within-group variability (i.e., the variability not explained by the grouping factor). Significant p-values are marked in bold.

The results indicated no significant change in ADM MEP latency between baseline and follow-up assessments (β = 1.92, SE = 1.94, p = 0.328), suggesting that time alone does not significantly influence MEP latency in this cohort. Neither age (β = 0.13, SE = 0.12, p = 0.286) nor sex (β = 0.24, SE = 1.95, p = 0.902) had significant effects on ADM MEP latency, indicating that these factors do not substantially contribute to changes in MEP latency. MS disease duration also did not have a significant effect on ADM MEP latency (β = -0.01, SE = 0.18, p = 0.951), suggesting that disease progression is not associated with changes in latency in this sample.

The random effect for participants showed a variance of 0, indicating no inter-individual variability in baseline ADM MEP latency values within this model. The residual variance (σ² = 34.89) reflects moderate within-individual variability across time points, which is the primary source of variability in this model.

## 2.6. ADM MEP amplitude

Table S2.6. Results of the linear mixed model longitudinal analysis for ADM MEP amplitude


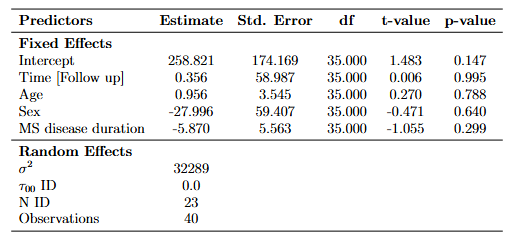


τ₀₀ (Tau), the variance of the random intercepts for the grouping factor (ID) representing the variability in the baseline levels between groups; σ², residual variance representing the within-group variability (i.e., the variability not explained by the grouping factor). Significant p-values are marked in bold.

The results indicated no significant change in ADM MEP amplitude between baseline and follow-up assessments (β = 0.37, SE = 58.99, p = 0.995), suggesting that time alone does not significantly influence motor evoked potential amplitude in this cohort. Neither age (β = 0.96, SE = 3.54, p = 0.788) nor sex (β = -27.99, SE = 59.41, p = 0.640) had significant effects on ADM MEP amplitude, indicating that these factors do not substantially contribute to changes in motor evoked potential amplitude. MS disease duration also did not have a significant effect on ADM MEP amplitude (β = -5.87, SE = 5.56, p = 0.299), suggesting that disease progression is not associated with changes in amplitude in this sample.

The random effect for participants showed a variance of 0, indicating no inter-individual variability in baseline ADM MEP amplitude values within this model. The residual variance (σ² = 32289) reflects the large within-individual variability across time points, which is the primary source of variability in this model.

## 2.7. TA RMT (%)

Table S2.7. Results of the linear mixed model longitudinal analysis for TA RMT (%)


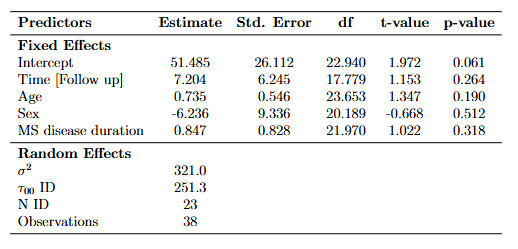


τ₀₀ (Tau), the variance of the random intercepts for the grouping factor (ID) representing the variability in the baseline levels between groups; σ², residual variance representing the within-group variability (i.e., the variability not explained by the grouping factor). Significant p-values are marked in bold.

The results indicated no significant change in TA RMT between baseline and follow-up assessments (β = 7.20, SE = 6.25, p = 0.264), suggesting that time alone does not significantly influence motor threshold in this cohort. Age did not have a significant effect on TA RMT (β = 0.74, SE = 5.55, p = 0.191), indicating that changes in age are not associated with motor threshold variability in this sample. Neither sex (β = -6.24, SE = 9.34, p = 0.512) nor MS disease duration (β = 0.85, SE = 0.83, p = 0.318) had significant effects on TA RMT, suggesting that these factors do not contribute to changes in motor threshold.

The random effect for participants, with a variance of 251.3, indicates substantial inter-individual variability in baseline TA RMT values. The residual variance (σ² = 321.0) reflects even larger within-individual variability across time points.

## 2.8. TA MEP latency

Table S2.8. Results of the linear mixed model longitudinal analysis for TA MEP latency


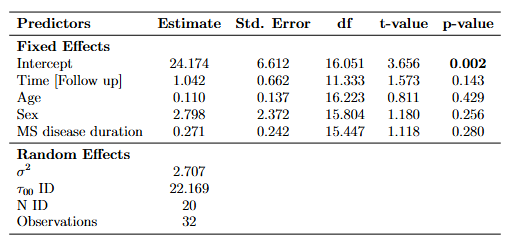


τ₀₀ (Tau), the variance of the random intercepts for the grouping factor (ID) representing the variability in the baseline levels between groups; σ², residual variance representing the within-group variability (i.e., the variability not explained by the grouping factor). Significant p-values are marked in bold.

The results indicated no significant change in TA MEP latency between baseline and follow-up assessments (β = 1.04, SE = 0.66, p = 0.143), suggesting that time alone does not significantly influence MEP latency in this cohort. Age did not have a significant effect on TA MEP latency (β = 0.11, SE = 0.14, p = 0.429), indicating that changes in age are not associated with motor evoked potential latency variability in this sample. Neither sex (β = 2.80, SE = 2.37, p = 0.256) nor MS disease duration (β = 0.27, SE = 0.24, p = 0.281) had significant effects on TA MEP latency, suggesting that these factors do not substantially contribute to changes in motor evoked potential latency.

The random effect for participants, with a variance of 22.17, indicates moderate inter-individual variability in baseline TA MEP latency values. The residual variance (σ² = 2.71) reflects small within-individual variability across time points.

## 2.9. TA MEP amplitude

Table S2.9. Results of the linear mixed model longitudinal analysis for TA MEP amplitude


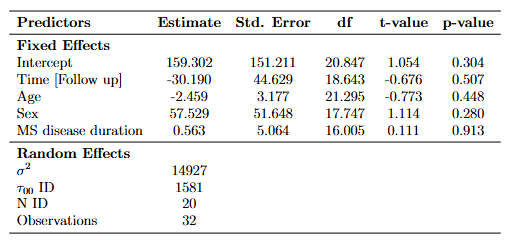


τ₀₀ (Tau), the variance of the random intercepts for the grouping factor (ID) representing the variability in the baseline levels between groups; σ², residual variance representing the within-group variability (i.e., the variability not explained by the grouping factor). Significant p-values are marked in bold.

The results indicated no significant change in TA MEP amplitude between baseline and follow-up assessments (β = -30.19, SE = 44.63, p = 0.507), suggesting that time alone does not significantly influence motor evoked potential amplitude in this cohort. Neither age (β = -2.46, SE = 3.18, p = 0.448) nor sex (β = 57.53, SE = 51.65, p = 0.280) had significant effects on TA MEP amplitude, indicating that these factors do not contribute to changes in motor evoked potential amplitude. MS disease duration also did not have a significant effect on TA MEP amplitude (β = 0.56, SE = 5.06, p = 0.913), suggesting that disease progression is not associated with changes in amplitude in this sample.

The random effect for participants, with a variance of 1581, indicates substantial inter-individual variability in baseline TA MEP amplitude values. The residual variance (σ² = 14927) reflects even larger within-individual variability across time points.

## 2.10. AH RMT (%)

Table S2.10. Results of the linear mixed model longitudinal analysis for AH RMT (%)


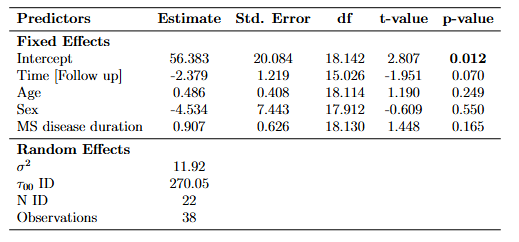


τ₀₀ (Tau), the variance of the random intercepts for the grouping factor (ID) representing the variability in the baseline levels between groups; σ², residual variance representing the within-group variability (i.e., the variability not explained by the grouping factor). Significant p-values are marked in bold.

The results indicated a non-significant reduction in AH RMT at follow-up compared to baseline (β = -2.38, SE = 1.22, p = 0.070). Age did not have a significant effect on AH RMT (β = 0.49, SE = 0.41, p = 0.249), indicating that changes in age are not associated with motor threshold variability in this sample. Neither sex (β = -4.53, SE = 7.44, p = 0.550) nor MS disease duration (β = 0.91, SE = 0.63, p = 0.165) had significant effects on AH RMT, suggesting that these factors do not contribute to changes in motor threshold in this cohort.

The random effect for participants, with a variance of 270.05, indicates substantial inter-individual variability in baseline AH RMT values. The residual variance (σ² = 11.92) reflects small within-individual variability across time points.

## 2.11. AH MEP latency

Table S2.11. Results of the linear mixed model longitudinal analysis for AH MEP latency


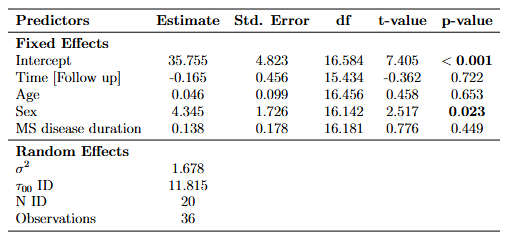


τ₀₀ (Tau), the variance of the random intercepts for the grouping factor (ID) representing the variability in the baseline levels between groups; σ², residual variance representing the within-group variability (i.e., the variability not explained by the grouping factor). Significant p-values are marked in bold.

The results indicated no significant change in AH MEP latency between baseline and follow-up assessments (β = -0.17, SE = 0.46, p = 0.722), suggesting that time alone does not significantly influence MEP latency in this cohort. Age did not have a significant effect on AH MEP latency (β = 0.05, SE = 0.10, p = 0.653), indicating that changes in age are not associated with motor evoked potential latency variability in this sample. However, sex was found to have a significant effect, with males having significantly higher AH MEP latency compared to females (β = 4.35, SE = 1.73, p = 0.023). MS disease duration did not have a significant effect on AH MEP latency (β = 0.14, SE = 0.18, p = 0.449), suggesting that disease progression is not associated with changes in latency in this sample.

The random effect for participants, with a variance of 11.82, indicates moderate inter-individual variability in baseline AH MEP latency values. The residual variance (σ² = 1.68) reflects small within-individual variability across time points.

## 2.12. AH MEP amplitude

Table S2.12. Results of the linear mixed model longitudinal analysis for AH MEP amplitude


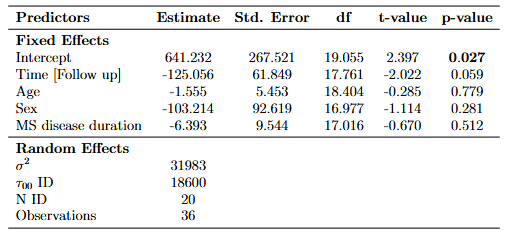


τ₀₀ (Tau), the variance of the random intercepts for the grouping factor (ID) representing the variability in the baseline levels between groups; σ², residual variance representing the within-group variability (i.e., the variability not explained by the grouping factor). Significant p-values are marked in bold.

The results indicated a marginally significant reduction in AH MEP amplitude at follow-up compared to baseline (β = -125.06, SE = 61.85, p = 0.059), suggesting a potential decline in motor evoked potential amplitude over time in this cohort. Age did not have a significant effect on AH MEP amplitude (β = -1.56, SE = 5.45, p = 0.779), indicating that changes in age are not associated with motor evoked potential amplitude variability in this sample. Neither sex (β = -103.21, SE = 92.62, p = 0.281) nor MS disease duration (β = -6.39, SE = 9.54, p = 0.512) had significant effects on AH MEP amplitude, suggesting that these factors do not contribute to changes in amplitude.

The random effect for participants, with a variance of 18600, indicates substantial inter-individual variability in baseline AH MEP amplitude values. The residual variance (σ² = 31983) reflects even larger within-individual variability across time points.
