# Supplementary material for: Correspondence of MRI and nTMS With EDSS in Multiple Sclerosis: Longitudinal Follow‐Up Study
Source: Ann Clin Transl Neurol. 2025 Apr 17;12(6):1240–55. doi: 10.1002/acn3.70041 (PMC12172135; doi:10.1002/acn3.70041)
Supplement: Supplementary file 2 — Supporting Information S2. [file ACN3-12-1240-s005.docx]

**Supplementary information S2**

**Detailed linear mixed model (LMM) results for Expanded Disability Status Scale (EDSS) parameters**

**Table of Contents**

[1. All relapsing-remitting multiple sclerosis (RRMS) participants 2](#_Toc181200272)

[1.1. EDSS 2](#_Toc181200273)

[1.2. EDSS Pyramid Score 3](#_Toc181200274)

[1.3. EDSS pyramid score right leg 4](#_Toc181200275)

[1.4. EDSS pyramid score left leg 5](#_Toc181200276)

[1.5. EDSS pyramid score right arm 6](#_Toc181200277)

[1.6. EDSS pyramid score left arm 7](#_Toc181200278)

[2. RRMS participants grouped based on their MEP latency findings (non-altered and altered MEP latency groups) 8](#_Toc181200279)

[2.1. EDSS 8](#_Toc181200280)

[2.2. EDSS Pyramid Score 9](#_Toc181200281)

[2.3. EDSS pyramid score right leg 10](#_Toc181200282)

[2.4. EDSS pyramid score left leg 11](#_Toc181200283)

[2.5. EDSS pyramid score right arm 12](#_Toc181200284)

[2.6. EDSS pyramid score left arm 13](#_Toc181200285)

# 1. All relapsing-remitting multiple sclerosis (RRMS) participants

A linear mixed-effects model was used to investigate the effects of time, age, sex, and MS disease duration on the Expanded Disability Status Scale (EDSS) parameters in individuals with multiple sclerosis. The models included time (baseline vs. follow-up) as a fixed effect, along with age, sex (coded as 1 = female, 2 = male), and MS disease duration as covariates. A random intercept for each participant was included to account for the correlation of repeated measures within the same individuals.

## 1.1. EDSS

Table S1.1. Results of the linear mixed model longitudinal analysis for EDSS


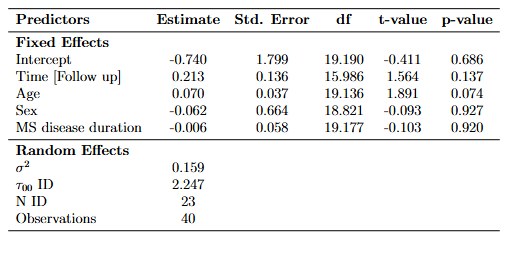


τ₀₀ (Tau), the variance of the random intercepts for the grouping factor (ID) representing the variability in the baseline levels between groups; σ², residual variance representing the within-group variability (i.e., the variability not explained by the grouping factor). Significant p-values are marked in bold.

The results indicated no significant change in EDSS between baseline and follow-up assessments (β = 0.21, SE = 0.14, p = 0.137), suggesting that time alone does not significantly influence disability status in this cohort. Age showed a trend towards significance (β = 0.07, SE = 0.04, p = 0.074), indicating a potential increase in disability with age, although this was not statistically significant. Neither sex (β = -0.06, SE = 0.66, p = 0.927) nor MS disease duration (β = -0.006, SE = 0.06, p = 0.919) showed significant effects on EDSS, suggesting that these factors do not contribute to changes in disability status over time in this cohort.

The random effect for participants, with a variance of 2.25, indicates small inter-individual variability in baseline EDSS values. The residual variance (σ² = 0.16) reflects relatively low within-individual variability across time points.

## 1.2. EDSS Pyramid Score

Table S1.2. Results of the linear mixed model longitudinal analysis for EDSS Pyramid Score


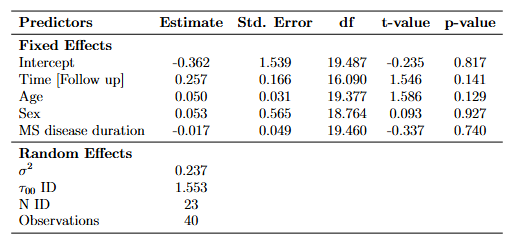


τ₀₀ (Tau), the variance of the random intercepts for the grouping factor (ID) representing the variability in the baseline levels between groups; σ², residual variance representing the within-group variability (i.e., the variability not explained by the grouping factor). Significant p-values are marked in bold.

The results showed no significant change in the EDSS Pyramid Score between baseline and follow-up assessments (β = 0.26, SE = 0.17, p = 0.141), indicating that time alone did not significantly influence the pyramid score in this cohort. Age also did not show a significant effect (β = 0.05, SE = 0.03, p = 0.129), suggesting that EDSS Pyramid Score may not be related to age in this sample. Similarly, sex (β = 0.05, SE = 0.56, p = 0.927) and MS disease duration (β = -0.01, SE = 0.05, p = 0.740) were not significant predictors, implying that these factors do not contribute to changes in the EDSS Pyramid Score over time.

The random effect for participants, with a variance of 1.55, indicates moderate inter-individual variability in baseline EDSS Pyramid Score values. The residual variance (σ² = 0.24) suggests a relatively low within-individual variability over time points.

## 1.3. EDSS pyramid score right leg

Table S1.3. Results of the linear mixed model longitudinal analysis for EDSS pyramid score right leg


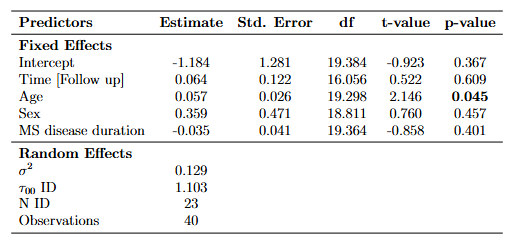


τ₀₀ (Tau), the variance of the random intercepts for the grouping factor (ID) representing the variability in the baseline levels between groups; σ², residual variance representing the within-group variability (i.e., the variability not explained by the grouping factor). Significant p-values are marked in bold.

The results indicated no significant change in the EDSS Pyramid Score for the right leg between baseline and follow-up assessments (β = 0.06, SE = 0.12, p = 0.609), suggesting that time alone does not significantly influence the pyramidal score for the right leg in this cohort. However, age showed a significant positive effect (β = 0.06, SE = 0.03, p = 0.045), indicating that older age is associated with higher EDSS Pyramid Scores for the right leg. Neither sex (β = 0.36, SE = 0.47, p = 0.457) nor MS disease duration (β = -0.04, SE = 0.04, p = 0.402) were significant predictors, implying that these factors do not impact the right leg pyramidal score over time.

The random effect for participants, with a variance of 1.10, indicates moderate inter-individual variability in baseline EDSS Pyramid Score for the right leg. The residual variance (σ² = 0.13) suggests relatively low within-individual variability over time points.

## 1.4. EDSS pyramid score left leg

Table S1.4. Results of the linear mixed model longitudinal analysis for EDSS pyramid score left leg


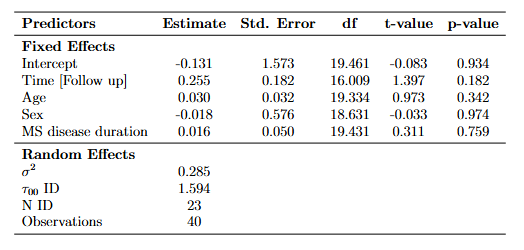


τ₀₀ (Tau), the variance of the random intercepts for the grouping factor (ID) representing the variability in the baseline levels between groups; σ², residual variance representing the within-group variability (i.e., the variability not explained by the grouping factor). Significant p-values are marked in bold.

The results indicated no significant change in the EDSS Pyramid Score for the left leg between baseline and follow-up assessments (β = 0.25, SE = 0.18, p = 0.182), suggesting that time alone does not significantly influence the pyramidal score for the left leg in this cohort. Similarly, neither age (β = 0.03, SE = 0.03, p = 0.342), sex (β = -0.02, SE = 0.58, p = 0.974), nor MS disease duration (β = 0.02, SE = 0.05, p = 0.759) showed significant effects on the EDSS Pyramid Score for the left leg. This indicates that these factors do not impact the left leg pyramidal score over time in this sample.

The random effect for participants, with a variance of 1.59, indicates moderate inter-individual variability in baseline EDSS Pyramid Score for the left leg. The residual variance (σ² = 0.29) suggests a low within-individual variability across time points.

## 1.5. EDSS pyramid score right arm

Table S1.5. Results of the linear mixed model longitudinal analysis for EDSS pyramid score right arm


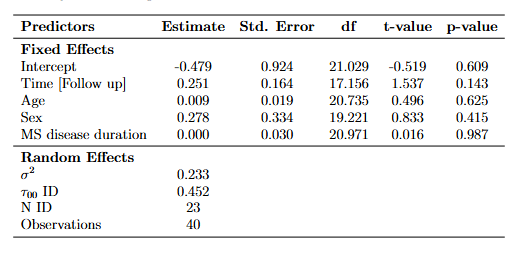


τ₀₀ (Tau), the variance of the random intercepts for the grouping factor (ID) representing the variability in the baseline levels between groups; σ², residual variance representing the within-group variability (i.e., the variability not explained by the grouping factor). Significant p-values are marked in bold.

The results indicated no significant change in the EDSS Pyramid Score for the right arm between baseline and follow-up assessments (β = 0.25, SE = 0.16, p = 0.143), suggesting that time alone does not significantly influence the pyramidal score for the right arm in this cohort. Similarly, neither age (β = 0.01, SE = 0.02, p = 0.625), sex (β = 0.28, SE = 0.33, p = 0.415), nor MS disease duration (β = 0.0005, SE = 0.03, p = 0.987) showed significant effects on the EDSS Pyramid Score for the right arm. This indicates that these variables do not impact the right arm pyramidal score over time in this sample.

The random effect for participants, with a variance of 0.45, indicates relatively low inter-individual variability in baseline EDSS Pyramid Score for the right arm. The residual variance (σ² = 0.23) also suggests low within-individual variability across time points.

## 1.6. EDSS pyramid score left arm

Table S1.6. Results of the linear mixed model longitudinal analysis for EDSS pyramid score left arm


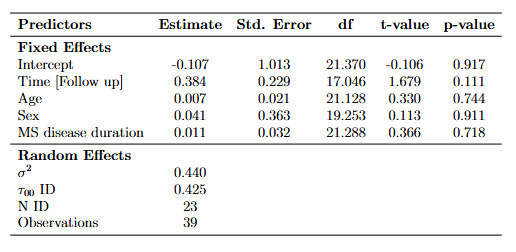


τ₀₀ (Tau), the variance of the random intercepts for the grouping factor (ID) representing the variability in the baseline levels between groups; σ², residual variance representing the within-group variability (i.e., the variability not explained by the grouping factor). Significant p-values are marked in bold.

The analysis did not show a significant change in the EDSS Pyramid Score for the left arm between baseline and follow-up (β = 0.38, SE = 0.23, p = 0.111), indicating that the pyramidal score for the left arm remained stable over time in this cohort. Additionally, age (β = 0.01, SE = 0.02, p = 0.744), sex (β = 0.04, SE = 0.36, p = 0.911), and MS disease duration (β = 0.01, SE = 0.03, p = 0.718) did not show significant effects on the EDSS Pyramid Score for the left arm, suggesting that these factors do not influence the pyramidal function in the left arm over the studied period.

The random effect for participants, with a variance of 0.43, indicates low inter-individual variability in the baseline EDSS Pyramid Score for the left arm. The residual variance (σ² = 0.44) also suggests a similar level of within-individual variability across time points.

# 2. RRMS participants grouped based on their MEP latency findings (non-altered and altered MEP latency groups)

A linear mixed-effects model (LMM) was fitted to investigate the effects of MEP latency grouping (non-altered vs. altered), time (baseline vs. follow-up), age, sex and MS disease duration on the Expanded Disability Status Scale (EDSS) parameters in individuals with multiple sclerosis. The model included MEP Group, time, and their interaction (MEP Group: Time) as fixed effects, along with age, MS disease duration and sex (coded as 1 = female, 2 = male) as covariates. A random intercept was included to account for repeated measures within participants (ID).

## 2.1. EDSS

Table S2.1. Results of the linear mixed model longitudinal analysis for EDSS


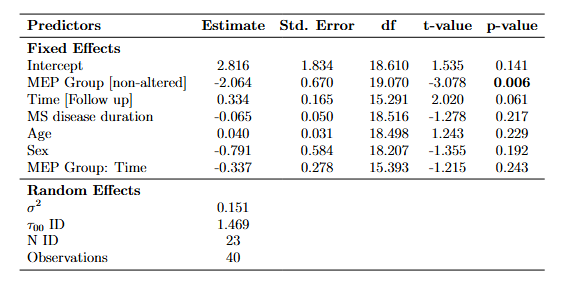


τ₀₀ (Tau), the variance of the random intercepts for the grouping factor (ID) representing the variability in the baseline levels between groups; σ², residual variance representing the within-group variability (i.e., the variability not explained by the grouping factor). Significant p-values are marked in bold.

The results indicated a significant difference in EDSS scores between the MEP latency altered and non-altered RRMS groups (β = -2.064, SE = 0.670, p = 0.006), with the non-altered MEP latency group having lower scores. Additionally, there was a marginally significant change in EDSS scores between baseline and follow-up (β = 0.334, SE = 0.165, p = 0.061), suggesting an increase over time. MS disease duration did not significantly impact EDSS scores (β = -0.065, SE = 0.050, p = 0.217). Age (β = 0.040, SE = 0.031, p = 0.229) and sex (β = -0.791, SE = 0.584, p = 0.192) were also not significant predictors.

The interaction between MEP Group and Time (β = -0.337, SE = 0.278, p = 0.243) was non-significant, indicating that changes in EDSS scores over time did not differ between the MEP latency altered and non-altered RRMS groups. The random intercept variance for participants was 1.469, reflecting differences in baseline EDSS scores between individuals, while the residual variance was 0.151, indicating within-individual variability over time.

## 2.2. EDSS Pyramid Score

Table S2.2. Results of the linear mixed model longitudinal analysis for EDSS Pyramid Score


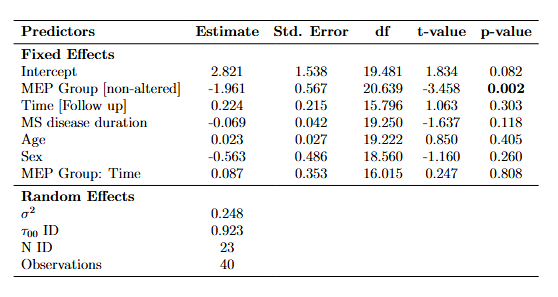


τ₀₀ (Tau), the variance of the random intercepts for the grouping factor (ID) representing the variability in the baseline levels between groups; σ², residual variance representing the within-group variability (i.e., the variability not explained by the grouping factor). Significant p-values are marked in bold.

The results indicated a significant difference in EDSS Pyramid Scores between the MEP latency altered and non-altered RRMS groups (β = -1.961, SE = 0.567, p = 0.002), with the non-altered MEP latency group having lower scores. The change in EDSS Pyramid Scores between baseline and follow-up (β = 0.224, SE = 0.215, p = 0.303) was not significant. MS disease duration did not significantly impact EDSS Pyramid Scores (β = -0.069, SE = 0.042, p = 0.118). Age (β = 0.023, SE = 0.027, p = 0.405) and sex (β = -0.563, SE = 0.486, p = 0.260) were also not significant predictors.

The interaction between MEP Group and Time (β = 0.087, SE = 0.353, p = 0.808) was non-significant, indicating that changes in EDSS Pyramid Scores over time did not differ between the MEP latency altered and non-altered RRMS groups. The random intercept variance for participants was 0.923, reflecting differences in baseline EDSS Pyramid Scores between individuals, while the residual variance was 0.248, indicating within-individual variability over time.

## 2.3. EDSS pyramid score right leg

Table S2.3. Results of the linear mixed model longitudinal analysis for EDSS pyramid score right leg


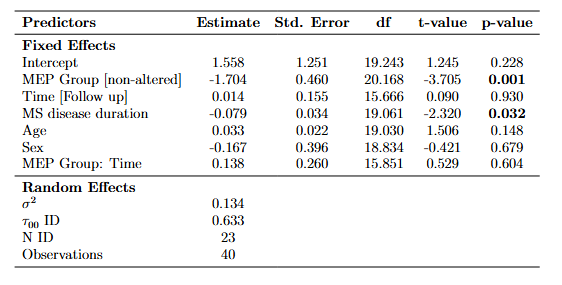


τ₀₀ (Tau), the variance of the random intercepts for the grouping factor (ID) representing the variability in the baseline levels between groups; σ², residual variance representing the within-group variability (i.e., the variability not explained by the grouping factor). Significant p-values are marked in bold.

The results indicated a significant difference in EDSS Pyramid Score between the MEP latency altered and non-altered RRMS groups (β = -1.704, SE = 0.460, p = 0.001), with the non-altered MEP latency group having lower scores. MS disease duration also significantly impacted EDSS Pyramid Score (β = -0.079, SE = 0.034, p = 0.032), showing a negative association. However, there was no significant change in EDSS Pyramid Score between baseline and follow-up (β = 0.014, SE = 0.155, p = 0.930). Age (β = 0.033, SE = 0.022, p = 0.148) and sex (β = -0.167, SE = 0.396, p = 0.679) were also not significant predictors.

The interaction between MEP Group and Time (β = 0.138, SE = 0.260, p = 0.604) was non-significant, indicating that changes in EDSS Pyramid Score over time did not differ between the MEP latency altered and non-altered RRMS groups. The random intercept variance for participants was 0.633, reflecting differences in baseline EDSS Pyramid Scores between individuals, while the residual variance was 0.134, indicating within-individual variability over time.

## 2.4. EDSS pyramid score left leg

Table S2.4. Results of the linear mixed model longitudinal analysis for EDSS pyramid score left leg


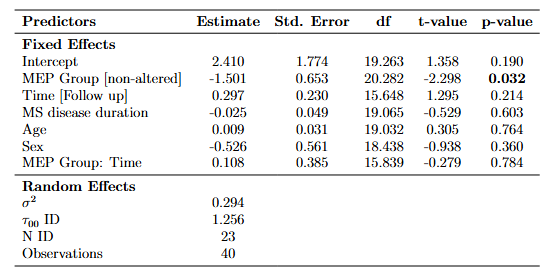


τ₀₀ (Tau), the variance of the random intercepts for the grouping factor (ID) representing the variability in the baseline levels between groups; σ², residual variance representing the within-group variability (i.e., the variability not explained by the grouping factor). Significant p-values are marked in bold.

The results showed a significant difference in EDSS Pyramid Scores between the MEP latency altered and non-altered groups (β = -1.501, SE = 0.653, p = 0.032), with the non-altered group having lower scores. The change in EDSS scores over time was not statistically significant (β = 0.297, SE = 0.230, p = 0.214), indicating no clear increase or decrease over the follow-up period. MS disease duration (β = -0.025, SE = 0.049, p = 0.603), age (β = 0.009, SE = 0.031, p = 0.764), and sex (β = -0.526, SE = 0.561, p = 0.360) were also not significant predictors of EDSS Pyramid Score.

The interaction between MEP Group and Time (β = 0.108, SE = 0.385, p = 0.784) was non-significant, suggesting that changes in EDSS scores over time did not differ between the altered and non-altered MEP latency groups. The random intercept variance for participants was 1.256, reflecting individual differences in baseline EDSS scores, while the residual variance was 0.294, indicating within-participant variability over time.

## 2.5. EDSS pyramid score right arm

Table S2.5. Results of the linear mixed model longitudinal analysis for EDSS pyramid score right arm


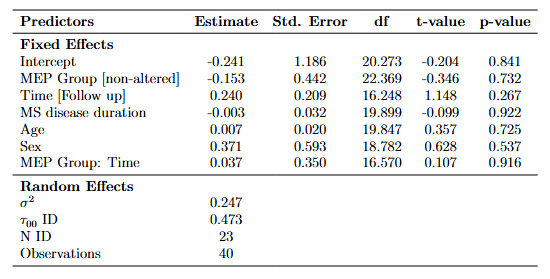


τ₀₀ (Tau), the variance of the random intercepts for the grouping factor (ID) representing the variability in the baseline levels between groups; σ², residual variance representing the within-group variability (i.e., the variability not explained by the grouping factor). Significant p-values are marked in bold.

The results showed no significant difference in EDSS Pyramid Scores between the non-altered and altered MEP latency groups (β = -0.153, SE = 0.442, p = 0.732). The change in EDSS scores over time was also not statistically significant (β = 0.240, SE = 0.209, p = 0.267), indicating minimal variation between baseline and follow-up. MS disease duration (β = -0.003, SE = 0.032, p = 0.922), age (β = 0.007, SE = 0.020, p = 0.725), and sex (β = 0.371, SE = 0.593, p = 0.537) were also not significant predictors.

The interaction between MEP Group and Time (β = 0.037, SE = 0.350, p = 0.916) was non-significant, indicating no differential effect over time based on MEP latency group. The random intercept variance for participants was 0.473, while the residual variance was 0.247, indicating the within-participant variability over time.

## 2.6. EDSS pyramid score left arm

Table S2.6. Results of the linear mixed model longitudinal analysis for EDSS pyramid score left arm


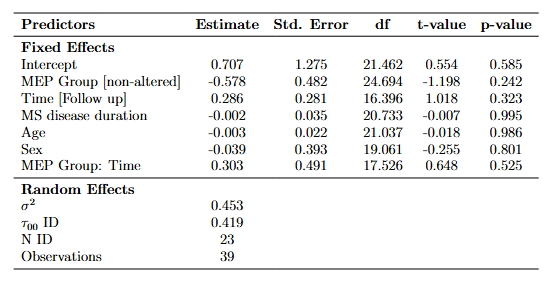


τ₀₀ (Tau), the variance of the random intercepts for the grouping factor (ID) representing the variability in the baseline levels between groups; σ², residual variance representing the within-group variability (i.e., the variability not explained by the grouping factor). Significant p-values are marked in bold.

The results showed no significant difference in EDSS Pyramid Scores between non-altered and altered MEP latency groups (β = -0.578, SE = 0.482, p = 0.242). The change in EDSS scores over time was also non-significant (β = 0.286, SE = 0.281, p = 0.323). MS disease duration (β = -0.002, SE = 0.035, p = 0.995), age (β = -0.003, SE = 0.022, p = 0.986), and sex (β = -0.039, SE = 0.393, p = 0.801) were not significant predictors.

The interaction between MEP Group and Time (β = 0.303, SE = 0.491, p = 0.525) was also non-significant, suggesting that changes in EDSS scores over time did not differ significantly based on MEP latency group. The random intercept variance for participants was 0.419, with a residual variance of 0.453, indicating variability within individuals over time.
