# Supplementary material for: Correspondence of MRI and nTMS With EDSS in Multiple Sclerosis: Longitudinal Follow‐Up Study
Source: Ann Clin Transl Neurol. 2025 Apr 17;12(6):1240–55. doi: 10.1002/acn3.70041 (PMC12172135; doi:10.1002/acn3.70041)
Supplement: Supplementary file 3 — Supporting Information S3. [file ACN3-12-1240-s001.docx]

**Supplementary information S3**

**Detailed (generalized) linear mixed model (GLMM / LMM) results for magnetic resonance imaging (MRI) parameters**

**Table of Contents**

[1. All relapsing-remitting multiple sclerosis (RRMS) participants 2](#_Toc181263101)

[1.1. Number of lesions 3](#_Toc181263102)

[1.2. Cortical 3](#_Toc181263103)

[1.3. Juxtacortical 4](#_Toc181263104)

[1.4. Periventricular 5](#_Toc181263105)

[1.5. Infratentorial 6](#_Toc181263106)

[1.6. Spinal cord 7](#_Toc181263107)

[1.7. Corpus callosum 8](#_Toc181263108)

[1.8. Number of lesions (all levels) 9](#_Toc181263109)

[1.9. Corticospinal tract Spine Right 10](#_Toc181263110)

[1.10. Corticospinal tract Spine Left 11](#_Toc181263111)

[1.11. Corticospinal tract brain Right 12](#_Toc181263112)

[1.12. Corticospinal tract brain Left 12](#_Toc181263113)

[1.13. Primary motor cortex Right 13](#_Toc181263114)

[1.14. Primary motor cortex Left 13](#_Toc181263115)

[2. RRMS participants grouped based on their MEP latency findings (non-altered and altered MEP latency groups) 14](#_Toc181263116)

[2.1. Number of lesions 14](#_Toc181263117)

[2.2. Cortical 15](#_Toc181263118)

[2.3. Juxtacortical 15](#_Toc181263119)

[2.4. Periventricular 16](#_Toc181263120)

[2.5. Infratentorial 17](#_Toc181263121)

[2.6. Spinal cord 18](#_Toc181263122)

[2.7. Corpus callosum 18](#_Toc181263123)

[2.8. Number of lesions (all levels) 19](#_Toc181263124)

[2.9. Corticospinal tract Spine Right 19](#_Toc181263125)

[2.10. Corticospinal tract Spine Left 19](#_Toc181263126)

[2.11. Corticospinal tract brain Right 19](#_Toc181263127)

[2.12. Corticospinal tract brain Left 19](#_Toc181263128)

[2.13. Primary motor cortex Right 20](#_Toc181263129)

[2.14. Primary motor cortex Left 20](#_Toc181263130)

# 1. All relapsing-remitting multiple sclerosis (RRMS) participants

A (generalized) linear mixed-effects models (GLMM/ LMM) was fitted to investigate the effects of time, age, and sex on each of the MRI parameters in individuals with multiple sclerosis. Each model included Time (Baseline vs. Follow-up) as a fixed effect, along with age and sex (coded as 1 = female, 2 = male) as covariates. A random intercept for each participant was included to account for the correlation of repeated measures within the same individuals.

For MRI parameters which represented count data (i.e., the number of lesions), the LMM was replaced by a generalized linear mixed model (GLMM) with a Poisson distribution, appropriate for modelling count data.

## 1.1. Number of lesions

Table S1.1. Results of the generalized linear mixed model (GLMM) longitudinal analysis for Number of lesions


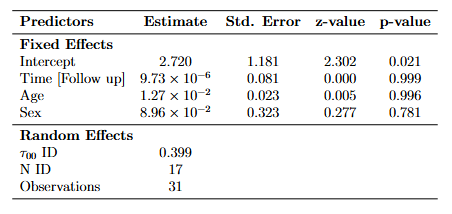


τ₀₀ (Tau), the variance of the random intercepts for the grouping factor (ID) representing the variability in the baseline levels between groups. Significant p-values are marked in bold.

There was no significant change in the number of lesions between baseline and follow-up assessments (β = 9.73×10^-6^, SE = 0.081, p = 0.999), suggesting that time alone does not significantly influence the number of lesions in this cohort. Age also did not have a significant effect on the number of lesions (β = 1.27×10^-2^, SE = 0.023, p = 0.996), indicating that changes in age are not associated with variability in lesion counts in this sample. The effect of sex was also not significant, with no significant difference in lesion counts between men and women (β = 8.96×10^-2^, SE = 0.323, p = 0.781).

The random effect for participants, with a variance of 0.399, indicates moderate inter-individual variability in baseline lesion counts.

## 1.2. Cortical

The GLMM did not converge.

## 1.3. Juxtacortical

Table S1.3. Results of the generalized linear mixed model (GLMM) longitudinal analysis for Juxtacortical number of lesions


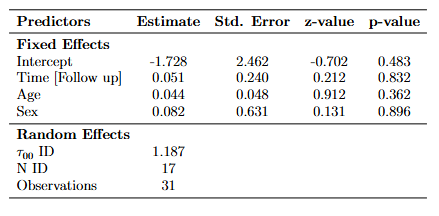


τ₀₀ (Tau), the variance of the random intercepts for the grouping factor (ID) representing the variability in the baseline levels between groups. Significant p-values are marked in bold.

The results indicated that there was no significant change in the Juxtacortical number of lesions between baseline and follow-up assessments (β = 0.051, SE = 0.240, p = 0.832), suggesting that time alone does not significantly influence the number of juxtacortical lesions in this cohort. Age also did not have a significant effect on the number of juxtacortical lesions (β = 0.044, SE = 0.049, p = 0.362), indicating that changes in age are not associated with variability in lesion counts in this sample. Similarly, the effect of sex was not significant, with no significant difference in the number of juxtacortical lesions between men and women (β = 0.083, SE = 0.631, p = 0.896).

The random effect for participants, with a variance of 1.187, indicates modest inter-individual variability in baseline juxtacortical lesion counts.

## 1.4. Periventricular

Table S1.4. Results of the generalized linear mixed model (GLMM) longitudinal analysis for Periventricular number of lesions


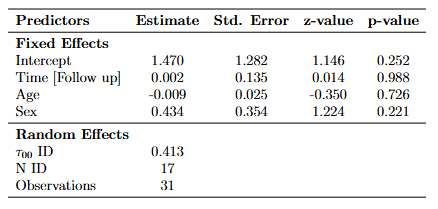


τ₀₀ (Tau), the variance of the random intercepts for the grouping factor (ID) representing the variability in the baseline levels between groups. Significant p-values are marked in bold.

The results indicated that there was no significant change in the Periventricular number of lesions between baseline and follow-up assessments (β = 0.002, SE = 0.135, p = 0.988), suggesting that time alone does not significantly influence the number of periventricular lesions in this cohort. Age also did not have a significant effect on the number of periventricular lesions (β = -0.009, SE = 0.025, p = 0.726), indicating that changes in age are not associated with variability in lesion counts in this sample. Similarly, the effect of sex was not significant, with no significant difference in the number of periventricular lesions between males and females (β = 0.434, SE = 0.354, p = 0.221).

The random effect for participants, with a variance of 0.413, indicates modest inter-individual variability in baseline periventricular lesion counts.

## 1.5. Infratentorial

Table S1.5. Results of the generalized linear mixed model (GLMM) longitudinal analysis for Infratentorial number of lesions


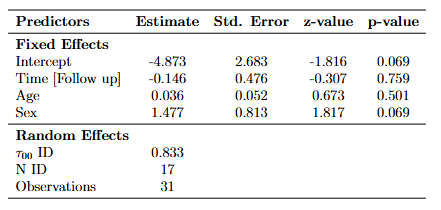


τ₀₀ (Tau), the variance of the random intercepts for the grouping factor (ID) representing the variability in the baseline levels between groups. Significant p-values are marked in bold.

The results indicated that there was no significant change in the infratentorial number of lesions between baseline and follow-up assessments (β = -0.146, SE = 0.476, p = 0.759), suggesting that time alone does not significantly influence the number of infratentorial lesions in this cohort. Age also did not have a significant effect on the number of infratentorial lesions (β = 0.036, SE = 0.052, p = 0.501), indicating that changes in age are not associated with variability in lesion counts in this sample. The effect of sex was also not significant, with no significant difference in the number of infratentorial lesions between men and women (β = 1.477, SE = 0.813, p = 0.069).

The random effect for participants, with a variance of 0.833, indicates minimal inter-individual variability in baseline infratentorial lesion counts.

## 1.6. Spinal cord

Table S1.6. Results of the generalized linear mixed model (GLMM) longitudinal analysis for Spinal cord number of lesions


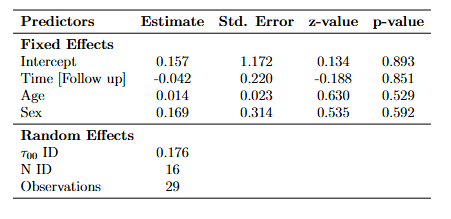


τ₀₀ (Tau), the variance of the random intercepts for the grouping factor (ID) representing the variability in the baseline levels between groups. Significant p-values are marked in bold.

The results indicated that there was no significant change in the Spinal cord number of lesions between baseline and follow-up assessments (β = -0.042, SE = 0.221, p = 0.851), suggesting that time alone does not significantly influence the number of spinal cord lesions in this cohort. Age also did not have a significant effect on the number of spinal cord lesions (β = 0.014, SE = 0.023, p = 0.529), indicating that changes in age are not associated with variability in lesion counts in this sample. Similarly, the effect of sex was not significant, with no significant difference in the number of spinal cord lesions between men and women (β = 0.168, SE = 0.314, p = 0.592).

The random effect for participants, with a variance of 0.176, indicates modest inter-individual variability in baseline spinal cord lesion counts.

## 1.7. Corpus callosum

Table S1.7. Results of the generalized linear mixed model (GLMM) longitudinal analysis for Corpus callosum number of lesions


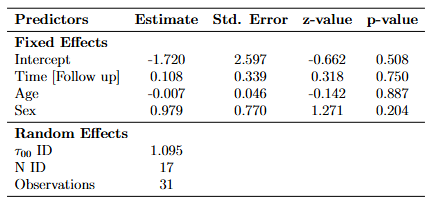


τ₀₀ (Tau), the variance of the random intercepts for the grouping factor (ID) representing the variability in the baseline levels between groups. Significant p-values are marked in bold.

The results indicated that there was no significant change in the Corpus callosum number of lesions between baseline and follow-up assessments (β = 0.108, SE = 0.339, p = 0.750), suggesting that time alone does not significantly influence the number of corpus callosum lesions in this cohort. Age also did not have a significant effect on the number of corpus callosum lesions (β = -0.007, SE = 0.046, p = 0.887), indicating that changes in age are not associated with variability in lesion counts in this sample. Similarly, the effect of sex was not significant, with no significant difference in the number of corpus callosum lesions between men and women (β = 0.979, SE = 0.771, p = 0.204).

The random effect for participants, with a variance of 1.095, indicates moderate inter-individual variability in baseline corpus callosum lesion counts.

## 1.8. Number of lesions (all levels)

Table S1.8. Results of the generalized linear mixed model (GLMM) longitudinal analysis for Number of lesions (all levels)


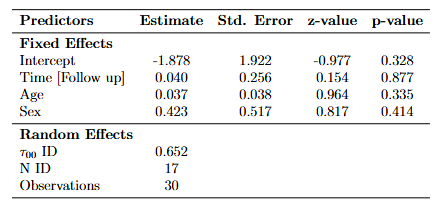


τ₀₀ (Tau), the variance of the random intercepts for the grouping factor (ID) representing the variability in the baseline levels between groups. Significant p-values are marked in bold.

The results indicated that there was no significant change in the number of lesions between baseline and follow-up assessments (β = 0.040, SE = 0.256, p = 0.877), suggesting that time alone does not significantly influence the number of lesions in this region for this cohort. Age also did not have a significant effect on the number of lesions (β = 0.037, SE = 0.038, p = 0.335), indicating that changes in age are not associated with variability in lesion counts in this sample. The effect of sex was also not significant, with no significant difference in the number of lesions between men and women (β = 0.423, SE = 0.517, p = 0.414).

The random effect for participants, with a variance of 0.652, indicates moderate inter-individual variability in baseline lesion counts.

## 1.9. Corticospinal tract Spine Right

Table S1.9. Results of the generalized linear mixed model (GLMM) longitudinal analysis for Corticospinal tract Spine Right


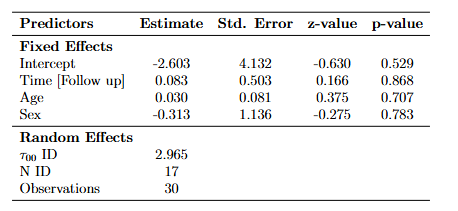


τ₀₀ (Tau), the variance of the random intercepts for the grouping factor (ID) representing the variability in the baseline levels between groups. Significant p-values are marked in bold.

The results indicated that there was no significant change in the number of Corticospinal tract lesions in the right spine between baseline and follow-up assessments (β = 0.083, SE = 0.503, p = 0.868), suggesting that time alone does not significantly influence the number of lesions in this region in this cohort. Age also did not have a significant effect on the number of Corticospinal tract lesions in the right spine (β = 0.030, SE = 0.081, p = 0.707), indicating that changes in age are not associated with variability in lesion counts in this sample. Similarly, the effect of sex was not significant, with no significant difference in the number of lesions between men and women (β = -0.313, SE = 1.137, p = 0.783).

The random effect for participants, with a variance of 2.965, indicates moderate inter-individual variability in baseline lesion counts of the Corticospinal tract in the right spine.

## 1.10. Corticospinal tract Spine Left

Table S1.10. Results of the generalized linear mixed model (GLMM) longitudinal analysis for Corticospinal tract Spine Left


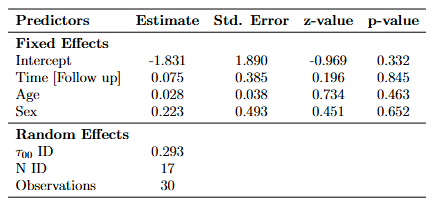


τ₀₀ (Tau), the variance of the random intercepts for the grouping factor (ID) representing the variability in the baseline levels between groups. Significant p-values are marked in bold.

The results indicated that there was no significant change in the number of Corticospinal tract lesions in the left spine between baseline and follow-up assessments (β = 0.076, SE = 0.385, p = 0.845), suggesting that time alone does not significantly influence the number of lesions in this region in this cohort. Age also did not have a significant effect on the number of Corticospinal tract lesions in the left spine (β = 0.028, SE = 0.038, p = 0.463), indicating that changes in age are not associated with variability in lesion counts in this sample. Similarly, the effect of sex was not significant, with no significant difference in the number of lesions between men and women (β = 0.223, SE = 0.493, p = 0.652).

The random effect for participants, with a variance of 0.293, indicates moderate inter-individual variability in baseline lesion counts of the Corticospinal tract in the left spine.

## 1.11. Corticospinal tract brain Right

The GLMM did not converge.

## 1.12. Corticospinal tract brain Left

Table S1.13. Results of the generalized linear mixed model (GLMM) longitudinal analysis for Corticospinal tract brain Left


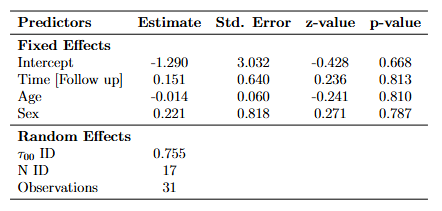


τ₀₀ (Tau), the variance of the random intercepts for the grouping factor (ID) representing the variability in the baseline levels between groups. Significant p-values are marked in bold.

The results indicated that there was no significant change in the number of Corticospinal tract lesions in the left between baseline and follow-up assessments (β = 0.151, SE = 0.640, p = 0.813), suggesting that time alone does not significantly influence the number of lesions in this region in this cohort. Age also did not have a significant effect on the number of Corticospinal tract lesions in the left brain (β = -0.014, SE = 0.060, p = 0.810), indicating that changes in age are not associated with variability in lesion counts in this sample. Similarly, the effect of sex was not significant, with no significant difference in the number of lesions between men and women (β = 0.221, SE = 0.818, p = 0.787).

The random effect for participants, with a variance of 0.755, indicates moderate inter-individual variability in baseline lesion counts of the Corticospinal tract in the left brain.

## 1.13. Primary motor cortex Right

Table S1.15. Results of the generalized linear mixed model (GLMM) longitudinal analysis for Primary motor cortex Right


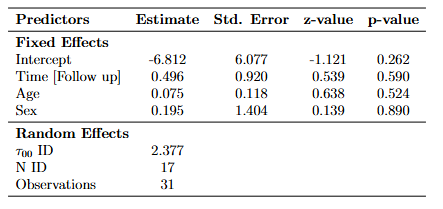


τ₀₀ (Tau), the variance of the random intercepts for the grouping factor (ID) representing the variability in the baseline levels between groups. Significant p-values are marked in bold.

The results indicated that there was no significant change in the number of lesions in the right primary motor cortex between baseline and follow-up assessments (β = 0.496, SE = 0.920, p = 0.590), suggesting that time alone does not significantly influence the number of lesions in this region in this cohort. Age also did not have a significant effect on the number of lesions in the right primary motor cortex (β = 0.075, SE = 0.118, p = 0.524), indicating that changes in age are not associated with variability in lesion counts in this sample. Similarly, the effect of sex was not significant, with no significant difference in the number of lesions between men and women (β = 0.195, SE = 1.404, p = 0.890).

The random effect for participants, with a variance of 2.377, indicates moderate inter-individual variability in baseline lesion counts of the right primary motor cortex.

## 1.14. Primary motor cortex Left

The GLMM did not converge.

# 2. RRMS participants grouped based on their MEP latency findings (non-altered and altered MEP latency groups)

A (generalized) linear mixed-effects model (GLMM/ LMM) was fitted to investigate the effects of MEP latency grouping (non-altered vs. altered), time (baseline vs. follow-up), age, and sex on the number of lesions. The model included MEP Group, Time, and their interaction (MEP Group: Time) as fixed effects, along with age and sex (coded as 1 = female, 2 = male) as covariates. A random intercept was included to account for repeated measures within participants (ID).

For MRI parameters which represented count data (i.e., the number of lesions), the LMM was replaced by a generalized linear mixed model (GLMM) with a Poisson distribution, appropriate for modelling count data.

## 2.1. Number of lesions

Table S2.1. Results of the generalized linear mixed model (GLMM) longitudinal analysis for Number of lesions


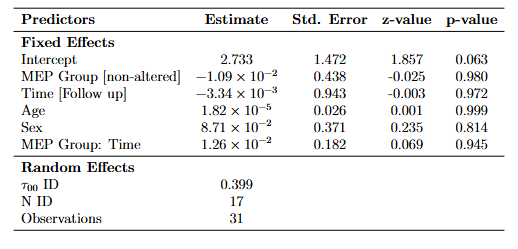


τ₀₀ (Tau), the variance of the random intercepts for the grouping factor (ID) representing the variability in the baseline levels between groups. Significant p-values are marked in bold.

There was no significant difference in the number of lesions between the pathological and normal MEP groups (β = -1.09×10^-2^, SE = 0.438, p = 0.980). Similarly, no significant change in the number of lesions over time was observed (β = -3.34×10^-3^, SE = 0.943, p = 0.9712). Age (β = -1.82×10^-5^, SE = 0.026, p = 0.999) and sex (β = 8.71×10^-2^, SE = 0.371, p = 0.814) were not significant predictors.

Additionally, the interaction between MEP Group and TimeFo was not significant (β = 1.26×10^-2^, SE = 0.182, p = 0.945), indicating that the effect of time on the number of lesions did not differ between the MEP latency groups.

The random intercept variance for participants was 0.399, reflecting differences in baseline lesion counts between individuals.

## 2.2. Cortical

The GLMM did not converge.

## 2.3. Juxtacortical

Table S2.3. Results of the generalized linear mixed model (GLMM) longitudinal analysis for Juxtacortical number of lesions


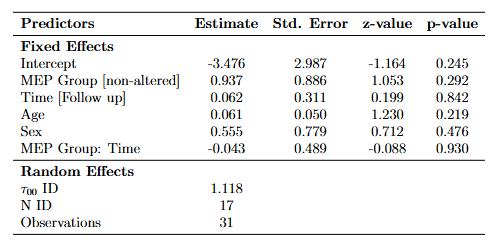


τ₀₀ (Tau), the variance of the random intercepts for the grouping factor (ID) representing the variability in the baseline levels between groups. Significant p-values are marked in bold.

There was no significant difference in the number of lesions between the altered and non-altered MEP groups (β = 0.937, SE = 0.886, p = 0.292). Additionally, no significant change in the number of lesions over time was observed (β = 0.062, SE = 0.311, p = 0.842). Age (β = 0.061, SE = 0.050, p = 0.219) and sex (β = 0.555, SE = 0.779, p = 0.476) were also not significant predictors.

The interaction between MEP Group and Time was not significant (β = -0.043, SE = 0.489, p = 0.930), indicating that the effect of time on the number of juxtacortical lesions did not differ between the MEP latency groups.

## 2.4. Periventricular

Table S2.4. Results of the generalized linear mixed model (GLMM) longitudinal analysis for Periventricular number of lesions


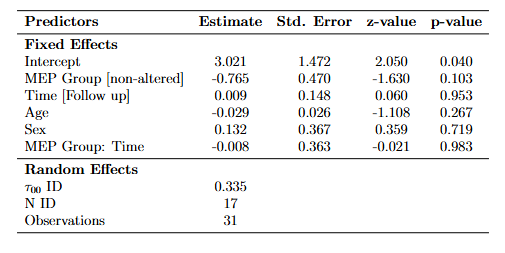


τ₀₀ (Tau), the variance of the random intercepts for the grouping factor (ID) representing the variability in the baseline levels between groups. Significant p-values are marked in bold.

There was no significant difference in the number of lesions between the altered and non-altered MEP groups (β = -0.765, SE = 0.470, p = 0.103). Similarly, no significant change in the number of lesions over time was observed (β = 0.009, SE = 0.148, p = 0.953). Age (β = -0.029, SE = 0.026, p = 0.267) and sex (β = 0.132, SE = 0.367, p = 0.719) were not significant predictors.

Additionally, the interaction between MEP group and Time was not significant (β = -0.008, SE = 0.363, p = 0.983), indicating that the effect of time on the number of periventricular lesions did not differ between the MEP groups.

The random intercept variance for participants was 0.335, reflecting minor differences in baseline periventricular lesion counts between individuals.

## 2.5. Infratentorial

Table S2.5. Results of the generalized linear mixed model (GLMM) longitudinal analysis for Infratentorial number of lesions


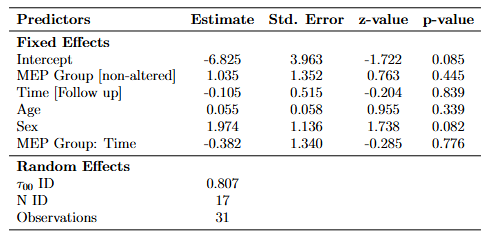


τ₀₀ (Tau), the variance of the random intercepts for the grouping factor (ID) representing the variability in the baseline levels between groups. Significant p-values are marked in bold.

There was no significant difference in the number of lesions between the altered and non-altered MEP groups (β = 1.035, SE = 1.352, p = 0.445). Similarly, no significant change in the number of lesions over time was observed (β = -0.105, SE = 0.515, p = 0.839). Age (β = 0.055, SE = 0.058, p = 0.339) and sex (β = 1.974, SE = 1.136, p = 0.082) were also not significant predictors.

Additionally, the interaction between MEP group and Time was not significant (β = -0.382, SE = 1.340, p = 0.776), indicating that the effect of time on the number of infratentorial lesions did not differ between the MEP latency groups.

## 2.6. Spinal cord

The GLMM did not converge.

## 2.7. Corpus callosum

Table S2.7. Results of the generalized linear mixed model (GLMM) longitudinal analysis for Corpus callosum number of lesions


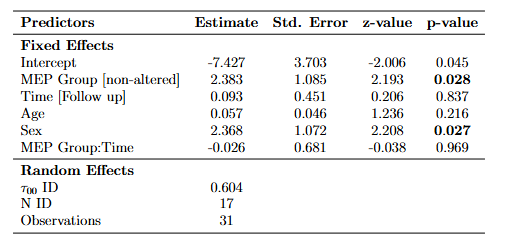


τ₀₀ (Tau), the variance of the random intercepts for the grouping factor (ID) representing the variability in the baseline levels between groups. Significant p-values are marked in bold.

There was a significant difference in the number of lesions between the altered and non-altered MEP groups (β = 2.383, SE = 1.085, p = 0.028), indicating that the pathological group had less lesions compared to the normal group. However, no significant change in the number of lesions over time was observed (β = 0.093, SE = 0.451, p = 0.837). Age (β = 0.057, SE = 0.046, p = 0.216) was not a significant predictor, while sex showed a significant effect (β = 2.368, SE = 1.072, p = 0.027).

The interaction between MEP Group and Time was not significant (β = -0.026, SE = 0.681, p = 0.969), indicating that the effect of time on the number of corpus callosum lesions did not differ between the MEP latency groups.

The random intercept variance for participants was 0.604, reflecting minor differences in baseline corpus callosum lesion counts between individuals.

## 2.8. Number of lesions (all levels)

Table S2.8. Results of the generalized linear mixed model (GLMM) longitudinal analysis for Number of lesions (all levels)


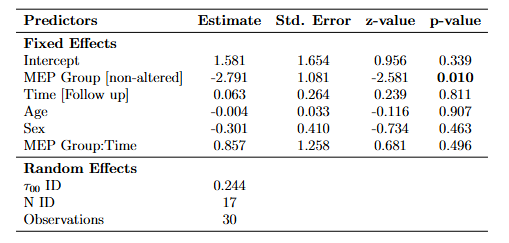


τ₀₀ (Tau), the variance of the random intercepts for the grouping factor (ID) representing the variability in the baseline levels between groups. Significant p-values are marked in bold.

There was a significant difference in the number of lesions between the altered and non-altered MEP groups (β = -2.791, SE = 1.081, p = 0.010), with the pathological group having more lesions. No significant change in the number of lesions over time was observed (β = 0.063, SE = 0.264, p = 0.811). Age (β = -0.004, SE = 0.033, p = 0.907) and sex (β = -0.301, SE = 0.410, p = 0.463) were also not significant predictors.

The interaction between MEP group and Time was not significant (β = 0.857, SE = 1.258, p = 0.496), indicating that the effect of time on the number of lesions in the periventricular region did not differ between the MEP latency groups.

## 2.9. Corticospinal tract Spine Right

The GLMM did not converge.

## 2.10. Corticospinal tract Spine Left

The GLMM did not converge.

## 2.11. Corticospinal tract brain Right

The GLMM did not converge.

## 2.12. Corticospinal tract brain Left

The GLMM did not converge.

## 2.13. Primary motor cortex Right

The GLMM did not converge.

## 2.14. Primary motor cortex Left

The GLMM did not converge.
