# Supplementary material for: Correspondence of MRI and nTMS With EDSS in Multiple Sclerosis: Longitudinal Follow‐Up Study
Source: Ann Clin Transl Neurol. 2025 Apr 17;12(6):1240–55. doi: 10.1002/acn3.70041 (PMC12172135; doi:10.1002/acn3.70041)
Supplement: Supplementary file 4 — Supporting Information S4. [file ACN3-12-1240-s003.docx]

**Supplementary information S4**

**Detailed results of correspondence of TMS and MRI data with EDSS scores**

**Table of Contents**

[1. ALL RRMS subjects 1](#_Toc181201068)

[2. RRMS participants grouped based on their MEP latency findings (non-altered and altered MEP latency groups) 1](#_Toc181201069)

[2.1. MRI-EDSS right leg 2](#_Toc181201070)

[2.2. MRI-EDSS left leg 2](#_Toc181201071)

[2.3. MRI-EDSS right arm 2](#_Toc181201072)

[2.4. MRI-EDSS left arm 2](#_Toc181201073)

# 1. ALL RRMS subjects

Table S1.1. Results of the McNemar’s test for the correspondence of TMS and MRI data with EDSS scores between baseline and follow-up

| **Correspondence** | **McNemar’s chi-squared statistic** | **df** | **p-value** |
| --- | --- | --- | --- |
| **TMS with EDSS** | | | |
| EDSS_TMS_right_leg | 0 | 1 | 1 |
| EDSS_TMS_left_leg | 0 | 1 | 1 |
| EDSS_TMS_right_arm | 0 | 1 | 1 |
| EDSS_TMS_left_arm | 0 | 1 | 1 |
|  |  |  |  |
| **MRI with EDSS** |  |  |  |
| MRI_EDSS_right_leg | 0 | 1 | 1 |
| MRI_EDSS_left_leg | NA | 1 | NA |
| MRI_EDSS_right_arm | 0 | 1 | 1 |
| MRI_EDSS_left_arm | 0 | 1 | 1 |

# 2. RRMS participants grouped based on their MEP latency findings (non-altered and altered MEP latency groups)

A Fisher's Exact Test was conducted to compare the correspondence of EDSS with MRI measures between MEP latency groups at baseline and at follow-up separately.

## 2.1. MRI-EDSS right leg

The test for baseline indicated no significant difference between the MEP groups (p = 1), with an odds ratio of 0.8655 and a 95% confidence interval ranging from 0.0645 to 14.66.

Similarly, the follow-up test also showed no significant difference (p = 0.2168), with an odds ratio of 0.1195 and a 95% confidence interval ranging from 0.0016 to 2.38.

These results suggest that there is no significant association between MEP grouping and the correspondence between MRI and EDSS right leg measures at either time point.

## 2.2. MRI-EDSS left leg

The test for baseline indicated no significant difference between the MEP groups (p = 0.299), with an odds ratio of 0.2754 and a 95% confidence interval ranging from 0.0151 to 3.70.

Similarly, the follow-up test also showed no significant difference (p = 0.2657), with an odds ratio of 0.1936 and a 95% confidence interval ranging from 0.0027 to 3.64.

These results suggest that there is no significant association between MEP grouping and the correspondence between MRI and EDSS left leg measures at either time point.

## 2.3. MRI-EDSS right arm

The test for baseline indicated no significant difference between the MEP groups (p = 0.119). The 95% confidence interval ranged from 0.4633 to infinity.

Similarly, the follow-up test also showed no significant difference (p = 1), with an odds ratio of 0.5286 and a 95% confidence interval ranging from 0.0250 to 10.81.

These results suggest that there is no significant association between MEP grouping and the correspondence between MRI and EDSS right arm measures at either time point.

## 2.4. MRI-EDSS left arm

The test for baseline indicated a significant difference between the MEP groups (p = 0.036), with an odds ratio of 14.00 and a 95% confidence interval ranging from 0.8522 to 972.43.

Conversely, the follow-up test showed no significant difference (p = 1), with an odds ratio of 1.90 and a 95% confidence interval ranging from 0.0925 to 39.99.

These results imply that while a significant association between MEP grouping and the correspondence between MRI and EDSS left arm measures was found at baseline, no such association was observed at the follow-up stage.
