# Supplementary material for: Correspondence of MRI and nTMS With EDSS in Multiple Sclerosis: Longitudinal Follow‐Up Study
Source: Ann Clin Transl Neurol. 2025 Apr 17;12(6):1240–55. doi: 10.1002/acn3.70041 (PMC12172135; doi:10.1002/acn3.70041)
Supplement: Supplementary file 5 — Supporting Information S5. [file ACN3-12-1240-s002.docx]

**Supplementary information S5**

**Detailed linear mixed model (LMM) results for DASS-21 and MSIS-29**

**Table of Contents**

[1. All relapsing-remitting multiple sclerosis (RRMS) participants 2](#_Toc181183884)

[1.1. DASS21 depression score 2](#_Toc181183885)

[1.2. DASS21 anxiety score 3](#_Toc181183886)

[1.3. DASS21 stress score 4](#_Toc181183887)

[1.4. MSIS29 physical score 5](#_Toc181183888)

[1.5. MSIS29 psychological score 6](#_Toc181183889)

[2. RRMS participants grouped based on their MEP latency findings (non-altered and altered MEP latency groups) 7](#_Toc181183890)

[2.1. DASS21 depression score 7](#_Toc181183891)

[2.2. DASS21 anxiety score 8](#_Toc181183892)

[2.3. DASS21 stress score 9](#_Toc181183893)

[2.4. MSIS29 physical score 10](#_Toc181183894)

[2.2. MSIS29 psychological score 11](#_Toc181183895)

# 1. All relapsing-remitting multiple sclerosis (RRMS) participants

## 1.1. DASS21­ depression score

Table S1.1. Results of the linear mixed model longitudinal analysis for DASS21­ depression score


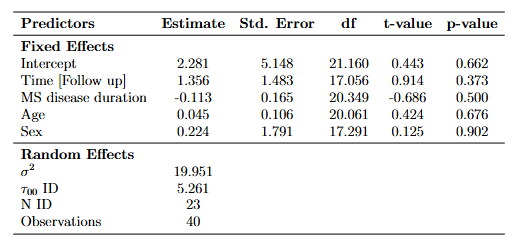


τ₀₀ (Tau), the variance of the random intercepts for the grouping factor (ID) representing the variability in the baseline levels between groups; σ², residual variance representing the within-group variability (i.e., the variability not explained by the grouping factor). Significant p-values are marked in bold.

A linear mixed-effects model was used to investigate the effects of time, MS disease duration, age, and sex on DASS21 depression scores in individuals. The model included time as a fixed effect, along with MS disease duration, age, and sex (coded as 1 = female, 2 = male) as covariates, with a random intercept for each participant to account for repeated measures within individuals.

The results showed no significant effect of time on DASS21 depression scores (β = 1.36, SE = 1.48, p = 0.373), indicating that time did not significantly influence depression scores in this cohort. Additionally, MS disease duration (β = -0.11, SE = 0.16, p = 0.500), age (β = 0.04, SE = 0.10, p = 0.676), and sex (β = 0.23, SE = 1.79, p = 0.902) were not significant predictors, suggesting that these variables did not significantly contribute to changes in depression scores over time.

The random effect for participants showed a variance of 5.26, suggesting moderate inter-individual variability, while the residual variance was 19.95, indicating considerable within-individual variability.

## 1.2. DASS21 anxiety score

Table S1.2. Results of the linear mixed model longitudinal analysis for DASS21 anxiety score


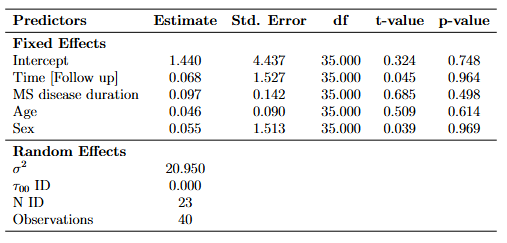


τ₀₀ (Tau), the variance of the random intercepts for the grouping factor (ID) representing the variability in the baseline levels between groups; σ², residual variance representing the within-group variability (i.e., the variability not explained by the grouping factor). Significant p-values are marked in bold.

A linear mixed-effects model was used to examine the effects of time, MS disease duration, age, and sex on DASS21 anxiety scores in individuals. The model included time as a fixed effect, along with MS disease duration, age, and sex, with a random intercept for each participant to account for within-individual correlations.

The results showed no significant effect of time on anxiety scores (β = 0.07, SE = 1.50, p = 0.964), indicating that time did not influence anxiety levels in this sample. Additionally, MS disease duration (β = 0.10, SE = 0.14, p = 0.498), age (β = 0.05, SE = 0.11, p = 0.614), and sex (β = 0.06, SE = 1.51, p = 0.969) were not significant predictors, suggesting that these variables did not significantly affect anxiety scores over time.

The random effect variance for participants was zero, meaning there was no inter-individual variability in baseline anxiety scores, while the residual variance was 20.95, reflecting considerable within-individual variability.

## 1.3. DASS21 stress score

Table S1.3. Results of the linear mixed model longitudinal analysis for DASS21 stress score


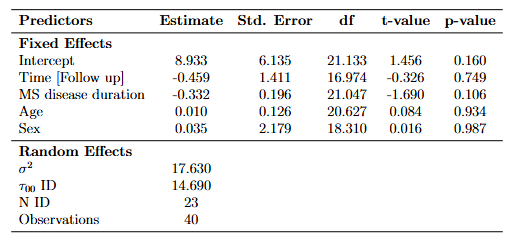


τ₀₀ (Tau), the variance of the random intercepts for the grouping factor (ID) representing the variability in the baseline levels between groups; σ², residual variance representing the within-group variability (i.e., the variability not explained by the grouping factor). Significant p-values are marked in bold.

A linear mixed-effects model was used to examine the effects of time, MS disease duration, age, and sex on DASS21 stress scores in individuals. The model included time as a fixed effect, along with MS disease duration, age, and sex, with a random intercept for each participant to account for within-individual correlations.

The results indicated no significant effect of time on stress scores (β = -0.46, SE = 1.41, p = 0.749), suggesting that time did not significantly influence stress levels in this sample. Additionally, MS disease duration (β = -0.33, SE = 0.20, p = 0.106), age (β = 0.01, SE = 0.13, p = 0.934), and sex (β = 0.03, SE = 2.18, p = 0.987) were not significant predictors, indicating that these variables did not affect stress scores over time.

The random effect variance for participants was 14.69, indicating moderate inter-individual variability, while the residual variance was 17.63, reflecting substantial within-individual variability.

## 1.4. MSIS29 physical score

Table S1.4. Results of the linear mixed model longitudinal analysis for MSIS29 physical score


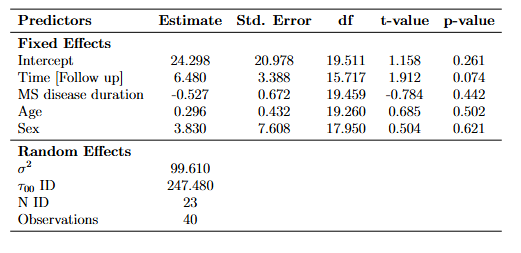


τ₀₀ (Tau), the variance of the random intercepts for the grouping factor (ID) representing the variability in the baseline levels between groups; σ², residual variance representing the within-group variability (i.e., the variability not explained by the grouping factor). Significant p-values are marked in bold.

A linear mixed-effects model was used to examine the effects of time, MS disease duration, age, and sex on MSIS29 physical scores in individuals. The model included time as a fixed effect, along with MS disease duration, age, and sex, with a random intercept for each participant to account for repeated measures.

The results indicated no significant effect of time on MSIS29 physical scores (β = 3.39, SE = 1.76, p = 0.074), suggesting time did not significantly influence physical scores in this sample. MS disease duration (β = -0.53, SE = 0.67, p = 0.442), age (β = 0.30, SE = 0.43, p = 0.501), and sex (β = 3.83, SE = 7.61, p = 0.621) were also not significant predictors, indicating that these factors did not affect physical scores over time.

The random effect variance for participants was 247.48, indicating substantial inter-individual variability, while the residual variance was 99.61, reflecting within-individual variability. Overall, none of the predictors had a significant effect on physical scores in this cohort.

## 1.5. MSIS29 psychological score

Table S1.5. Results of the linear mixed model longitudinal analysis for MSIS29 psychological score


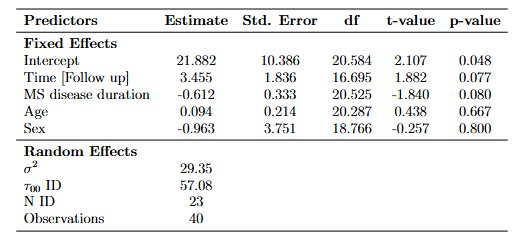


τ₀₀ (Tau), the variance of the random intercepts for the grouping factor (ID) representing the variability in the baseline levels between groups; σ², residual variance representing the within-group variability (i.e., the variability not explained by the grouping factor). Significant p-values are marked in bold.

A linear mixed-effects model was used to investigate the effects of time, MS disease duration, age, and sex on MSIS29 psychological scores in individuals. The model included time as a fixed effect, along with MS disease duration, age, and sex, with a random intercept for each participant to account for repeated measures.

The results indicated no significant effect of time on psychological scores (β = 3.45, SE = 1.84, p = 0.077). MS disease duration (β = -0.61, SE = 0.33, p = 0.080), age (β = 0.09, SE = 0.21, p = 0.667), and sex (β = -0.96, SE = 3.75, p = 0.800) were also not significant predictors.

The random effect variance for participants was 57.08, indicating moderate inter-individual variability, while the residual variance was 29.35, reflecting within-individual variability. Overall, none of the predictors had a statistically significant effect on psychological scores in this cohort.

# 2. RRMS participants grouped based on their MEP latency findings (non-altered and altered MEP latency groups)

## 2.1. DASS21 depression score

Table S2.1. Results of the linear mixed model longitudinal analysis for DASS21 depression score


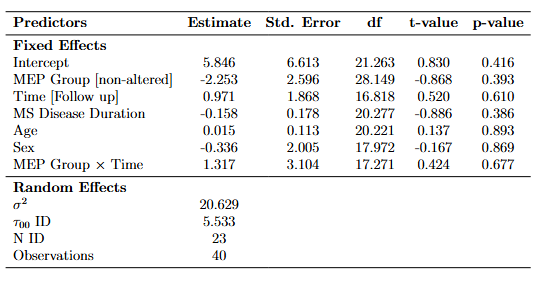


τ₀₀ (Tau), the variance of the random intercepts for the grouping factor (ID) representing the variability in the baseline levels between groups; σ², residual variance representing the within-group variability (i.e., the variability not explained by the grouping factor). Significant p-values are marked in bold.

A linear mixed-effects model (LMM) was conducted to investigate the effects of MEP group (RRMS with non-altered and altered MEP latency findings), time (baseline vs. follow-up), and MS disease duration on DASS21 depression scores. The model included MEP Group, Time, MS disease duration, as well as their interaction (MEP Group: Time) as fixed effects, while adjusting for age and sex. A random intercept was included to account for repeated measures within participants.

The results indicated no significant difference in DASS21 depression scores between the RRMS with non-altered and altered MEP latency findings (β = -2.253, SE = 2.596, p = 0.393), nor was there a significant change in scores between baseline and follow-up assessments (β = 0.971, SE = 1.867, p = 0.610).

MS disease duration did not have a significant impact on depression scores (β = -0.158, SE = 0.178, p = 0.386), and neither age (β = 0.0155, SE = 0.113, p = 0.893) nor sex (β = -0.3356, SE = 2.0046, p = 0.869) were significant predictors.

Additionally, the interaction between MEP Group and Time (β = 1.317, SE = 3.104, p = 0.677) was not significant, indicating that changes in depression scores over time did not differ between the MEP groups. The random intercept variance for participants was 5.533, reflecting differences in baseline depression scores between individuals, while the residual variance was

20.629, indicating within-individual variability over time.

## 2.2. DASS21 anxiety score

Table S2.2. Results of the linear mixed model longitudinal analysis for DASS21 anxiety score


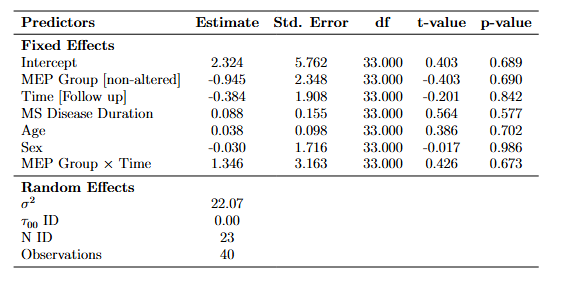


τ₀₀ (Tau), the variance of the random intercepts for the grouping factor (ID) representing the variability in the baseline levels between groups; σ², residual variance representing the within-group variability (i.e., the variability not explained by the grouping factor). Significant p-values are marked in bold.

A linear mixed-effects model (LMM) was conducted to investigate the effects of MEP group (RRMS with non-altered and altered MEP latency findings), time (baseline vs. follow-up), and MS disease duration on DASS21 anxiety scores. The model included MEP Group, Time, MS disease duration, and their interaction (MEP Group: Time) as fixed effects, while adjusting for age and sex. A random intercept was included to account for repeated measures within participants.

The results indicated no significant difference in DASS21 anxiety scores between the RRMS with non-altered and altered MEP latency findings (β = -0.945, SE = 2.348, p = 0.690), nor was there a significant change in scores over time (β = -0.384, SE = 1.908, p = 0.842). MS disease duration did not have a significant impact on anxiety scores (β = 0.088, SE = 0.155, p = 0.577), and neither age (β = 0.038, SE = 0.098, p = 0.702) nor sex (β = -0.030, SE = 1.716, p = 0.986) were significant predictors.

Additionally, the interaction between MEP Group and Time (β = 1.345, SE = 3.163, p = 0.673) was not significant, indicating that changes in anxiety scores over time did not differ between the MEP groups. The random intercept variance for participants was effectively zero, reflecting minimal variability in baseline anxiety scores between individuals, while the residual variance was 22.07, indicating variability within individuals over time.

## 2.3. DASS21 stress score

Table S2.3. Results of the linear mixed model longitudinal analysis for DASS21 stress score


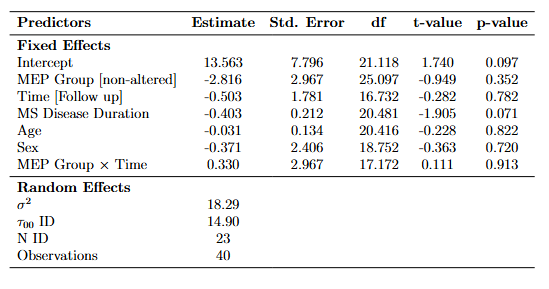


τ₀₀ (Tau), the variance of the random intercepts for the grouping factor (ID) representing the variability in the baseline levels between groups; σ², residual variance representing the within-group variability (i.e., the variability not explained by the grouping factor). Significant p-values are marked in bold.

A linear mixed-effects model (LMM) was conducted to investigate the effects of MEP group (RRMS with non-altered and altered MEP latency findings), time (baseline vs. follow-up), and MS disease duration on DASS21 stress scores. The model included MEP Group, Time, MS disease duration, and their interaction (MEP Group: Time) as fixed effects, while adjusting for age and sex. A random intercept was included to account for repeated measures within participants.

The results indicated no significant difference in DASS21 stress scores between the RRMS with non-altered and altered MEP latency findings (β = -2.816, SE = 2.967, p = 0.352), nor was there a significant change in scores between baseline and follow-up assessments (β = -0.503, SE = 1.781, p = 0.782). MS disease duration did not significantly impact stress scores (β = -0.036, SE = 0.212, p = 0.071). Additionally, age (β = -0.031, SE = 0.134, p = 0.822) and sex (β = -0.873, SE = 2.406, p = 0.720) were not significant predictors.

The interaction between MEP Group and Time (β = 0.330, SE = 2.967, p = 0.913) was also non-significant, indicating that changes in stress scores over time did not differ between the MEP groups. The random intercept variance for participants was 14.90, reflecting differences in baseline stress scores between individuals, while the residual variance was 18.29, indicating within-individual variability over time.

## 2.4. MSIS29 physical score

Table S2.4. Results of the linear mixed model longitudinal analysis for MSIS29 physical score


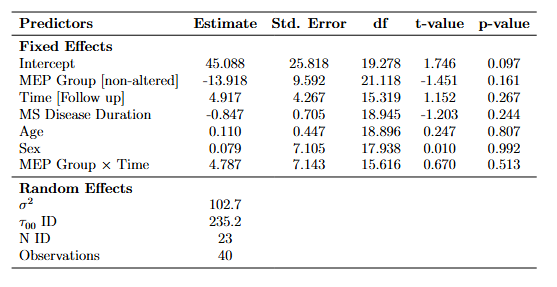


τ₀₀ (Tau), the variance of the random intercepts for the grouping factor (ID) representing the variability in the baseline levels between groups; σ², residual variance representing the within-group variability (i.e., the variability not explained by the grouping factor). Significant p-values are marked in bold.

A linear mixed-effects model (LMM) was conducted to investigate the effects of MEP group (RRMS with non-altered and altered MEP latency findings), time (baseline vs. follow-up), and MS disease duration on MSIS29 physical scores. The model included MEP Group, Time, MS disease duration, and their interaction (MEP Group: Time) as fixed effects, while adjusting for age and sex. A random intercept was included to account for repeated measures within participants.

The results indicated no significant difference in MSIS29 physical scores between the RRMS with non-altered and altered MEP latency findings (β = -13.918, SE = 9.592, p = 0.161), nor was there a significant change in scores between baseline and follow-up assessments (β = 4.917, SE = 4.267, p = 0.267). MS disease duration did not significantly impact physical scores (β = -0.847, SE = 0.705, p = 0.244). Additionally, age (β = 0.110, SE = 0.447, p = 0.807) and sex (β = 0.079, SE = 7.105, p = 0.992) were not significant predictors.

The interaction between MEP Group and Time (β = 4.787, SE = 7.143, p = 0.513) was also non-significant, indicating that changes in physical scores over time did not differ between the MEP groups. The random intercept variance for participants was 235.2, reflecting differences in baseline physical scores between individuals, while the residual variance was 102.7, indicating within-individual variability over time.

## 2.2. MSIS29 psychological score

Table S2.5. Results of the linear mixed model longitudinal analysis for MSIS29 psychological score


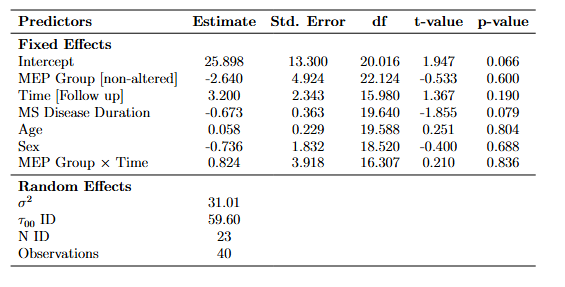


τ₀₀ (Tau), the variance of the random intercepts for the grouping factor (ID) representing the variability in the baseline levels between groups; σ², residual variance representing the within-group variability (i.e., the variability not explained by the grouping factor). Significant p-values are marked in bold.

A linear mixed-effects model (LMM) was conducted to investigate the effects of MEP group (RRMS with non-altered and altered MEP latency findings), time (baseline vs. follow-up), and MS disease duration on MSIS29 psychological scores. The model included MEP Group, Time, MS disease duration, and their interaction (MEP Group: Time) as fixed effects, while adjusting for age and sex. A random intercept was included to account for repeated measures within participants.

The results indicated no significant difference in MSIS29 psychological scores between the RRMS with non-altered and altered MEP latency findings (β = -2.640, SE = 4.924, p = 0.600), nor was there a significant change in scores between baseline and follow-up assessments (β = 3.200, SE = 2.343, p = 0.190). MS disease duration did not significantly impact psychological scores (β = -0.673, SE = 0.363, p = 0.079). Additionally, age (β = 0.058, SE = 0.229, p = 0.804) and sex (β = -0.736, SE = 1.832, p = 0.688) were not significant predictors.

The interaction between MEP Group and Time (β = 0.824, SE = 3.918, p = 0.836) was also non-significant, indicating that changes in psychological scores over time did not differ between the MEP groups. The random intercept variance for participants was 59.60, reflecting differences in baseline psychological scores between individuals, while the residual variance was 31.01, indicating within-individual variability over time.
